# Precise synthetic control of exclusive ligand effect boosts oxygen reduction catalysis

**DOI:** 10.1038/s41467-023-42514-w

**Published:** 2023-10-28

**Authors:** Lu Tao, Kai Wang, Fan Lv, Hongtian Mi, Fangxu Lin, Heng Luo, Hongyu Guo, Qinghua Zhang, Lin Gu, Mingchuan Luo, Shaojun Guo

**Affiliations:** 1 School of Materials Science and Engineering, Peking University, Beijing, 100871 China; 2 School of Materials Science and Engineering, University of Science and Technology Beijing, Beijing, 100083 China; 3 Beijing National Laboratory for Condensed Matter and Institute of Physics, Chinese Academy of Sciences, Beijing, 100190 China

**Keywords:** Fuel cells, Electronic properties and materials, Electrocatalysis

## Abstract

Ligand effect, induced by charge transfer between catalytic surface and substrate in core/shell structure, was widely proved to benefit Pt-catalyzed oxygen reduction reaction by tuning the position of *d*-band center of Pt theoretically. However, ligand effect is always convoluted by strain effect in real core/shell nanostructure; therefore, it remains experimentally unknown whether and how much the ligand effect solely contributes electrocatalytic activity improvements. Herein, we report precise synthesis of a kind of Pd_3_Ru_1_/Pt core/shell nanoplates with exclusive ligand effect for oxygen reduction reaction. Layer-by-layer growth of Pt overlayers onto Pd_3_Ru_1_ nanoplates can guarantee no lattice mismatch between core and shell because the well-designed Pd_3_Ru_1_ has the same lattice parameters as Pt. Electron transfer, due to the exclusive ligand effect, from Pd_3_Ru_1_ to Pt leads to a downshift of *d*-band center of Pt. The optimal Pd_3_Ru_1_/Pt_1-2L_ nanoplates achieve excellent activity and stability for oxygen reduction reaction in alkaline/acid electrolyte.

## Abstract (web summary)

Deconvoluting and quantitating ligand effect from the typical strain-ligand effects in a real catalytic structure remains challenging. Here, the authors report a core/shell catalyst model to quantitate how much ligand effect solely contributes to electrocatalytic performance through experimental design.

## Introduction

Oxygen reduction reaction (ORR), as the primary cathodic reaction in clean energy conversion/storage systems such as fuel cells and metal-air batteries, has been of great interest in recent few decades^1,2^. The ORR kinetic barriers and high cost of used Pt electrocatalysts are the major bottlenecks in the development of such sustainable energy devices^3,4^. Tremendous research efforts have been devoted to developing low-Pt electrocatalysts and unraveling the ORR mechanism for accelerating ORR^5–7^. It is well documented that the energy of the *d*-band center of the metal catalyst correlates to the adsorption energy, activation energy, and dissociation energy of small molecules^8^. Based on the acknowledged *d*-band center theory, the position of the *d*-band center of Pt determines the adsorption strength of the oxygenated intermediate on Pt active sites^9,10^. The core/shell structural engineering achieved by the deposition of a few Pt atomic layers, preferably monolayer, on a heterogeneous substrate, has been regarded as an effective strategy to tune the *d*-band center of surface Pt and optimize the adsorption strength based on the well-known strain and/or ligand effects^11–13^. In terms of the M/Pt core/shell models, strain effect on electrocatalytic activity can be well engineered by tuning the thickness of Pt shells or atomic composition, and the correlation of geometric strain, *d*-band center, adsorption strength, and catalytic activity of Pt has been studied^14,15^. However, short-range ligand effect, induced by charge transfer between surface Pt and sublayer heteroatoms in the core/shell nanostructures, usually has to be mixed with the strain effect because Pt shell and heterogeneous substrates always differ in lattice parameter^16–18^. This inevitable case in the core/shell nanostructures makes it very difficult to determine and quantify experimentally whether the exclusive ligand effect can greatly boost the intrinsic activity of Pt for ORR, though it is a basic assumption in theoretical calculations.

Herein, we report the precise synthesis of a class of Pd_3_Ru_1_ nanoplates (NPs) with the same lattice parameters as pure Pt for the growth of Pt atomic overlayers for well-revealing exclusive ligand effect for greatly enhancing the ORR electrocatalysis experimentally and computationally. X-ray absorption near-edge structure spectra, extended X-ray absorption fine structure spectra, X-ray photoelectron spectra results, and density functional theory (DFT) calculations reveal that the ligand effect caused by electronic donation from Pd_3_Ru_1_ to Pt can result in an increase of electron density in the 5*d* orbitals of Pt, thus the downshift of *d*-band center of Pt compared to the bulk Pt. The strong exclusive ligand effect from Pd_3_Ru_1_ core to monolayer Pt shell in Pd_3_Ru_1_/Pt_1-2L_ NPs makes them display remarkable mass activity (MA) of 10.3/4.59 amperes per milligram of Pt at 0.90 V *versus* reversible hydrogen electrode towards ORR in alkaline/acid electrolyte, 51.5/25.5 times higher than those of commercial Pt/C catalyst. Furthermore, Pd_3_Ru_1_/Pt_1-2L_ NPs can maintain 78.2%/52.1% of MA over 30,000 potential cycles in alkaline/acid electrolyte.

## Results

### Synthesis and structural characterizations

**Fig. 1:**
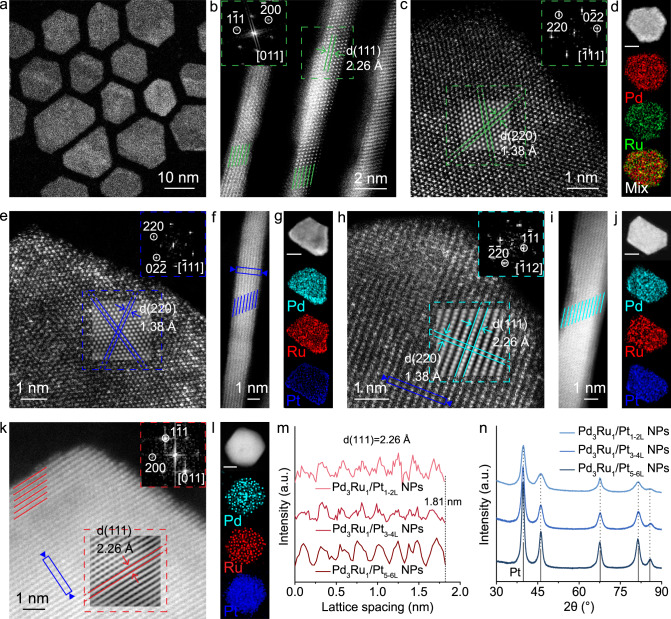
Structural analysis for Pd_3_Ru_1_ NPs and Pd_3_Ru_1_/Pt_nL_ NPs. **a** HAADF-STEM image of Pd_3_Ru_1_ NPs. **b**, **c** Atomic-resolution HAADF-STEM images of Pd_3_Ru_1_ NP with the corresponding FFT pattern taken from the green dashed area in (**b**, **c**), respectively. **d** EDS elemental mapping images of Pd_3_Ru_1_ NP. **e**, **f** Atomic-resolution HAADF-STEM images of Pd_3_Ru_1_/Pt_1-2L_ NPs with the corresponding FFT pattern taken from the blue dashed square in **e**. **g** EDS elemental mapping images of Pd_3_Ru_1_/Pt_1-2L_ NP. **h**, **i** Atomic-resolution HAADF-STEM images of Pd_3_Ru_1_/Pt_3-4L_ NPs with the corresponding FFT pattern taken from the cyan dashed square in **h**. **j** EDS elemental mapping images of Pd_3_Ru_1_/Pt_3-4L_ NP. **k** Atomic-resolution HAADF-STEM image of Pd_3_Ru_1_/Pt_5-6L_ NP with the corresponding FFT pattern taken from the red dashed square in (**k**). **l** EDS elemental mapping images of Pd_3_Ru_1_/Pt_5-6L_ NP. **m** Integrated pixel intensities of the Pd_3_Ru_1_/Pt_nL_ phases taken from the blue solid rectangles in (**f**, **h**, **k**), respectively. **n** PXRD patterns of Pd_3_Ru_1_/Pt_nL_ NPs. PDF card: Pt 04-0802. The scale bars in (**d**, **g**, **j**, **l**): 5 nm.

The Pd_*x*_Ru_1−*x*_ NPs were synthesized by using palladium diacetylacetonate (Pd(acac)_2_) and dodecacarbonyl triruthenium (Ru_3_(CO)_12_) as the metal precursors, oleylamine as the solvent and surfactant, and ascorbic acid as the reducing agent (details in Methods and Supplementary Table 1). The lattice parameters of Pd_*x*_Ru_1−*x*_ NPs could be adjusted by changing the elemental proportion of Pd to Ru (Supplementary Figs. 1 and 2). The crystal type and lattice spacings of Pd_3_Ru_1_ were found to be consistent with those of Pt. The transmission electron microscopy (TEM) images (Supplementary Fig. 3) and high-angle annular dark-field scanning TEM (HAADF-STEM) image (Fig. 1a) show that the as-synthesized Pd_3_Ru_1_ NPs have the hexagonal morphology with the lateral size of 10–20 nm. High-resolution HAADF-STEM results (Fig. 1b, c) reveal that the Pd_3_Ru_1_ NPs have an average thickness of 1.35 nm (equivalent to about 6 atomic layers thick, counted by green lines in Fig. 1b). The corresponding fast Fourier transform (FFT) patterns, taken from the green dashed regions in Fig. 1b, c, depict the typical characteristic diffraction pattern of [011] zone axes and [111] zone axes of the face-centered cubic (*fcc*) structure, respectively. Accordingly, the lattice spacings of (111) plane (Fig. 1b) and (220) plane (Fig. 1c), are calculated to be 2.26 Å and 1.38 Å, respectively, in consistent with those of Pt (111) and Pt (220) (Supplementary Fig. 4). The energy-dispersive X-ray spectroscopy (EDS) elemental mappings (Fig. 1d) of one typical Pd_3_Ru_1_ NP and corresponding line-scan profile (Supplementary Fig. 5a) across the NP reveal the entire-overlapping of Pd and Ru elemental signals in the NP, demonstrating the absence of composition segregation in the Pd_3_Ru_1_ phase. The element ratio of Pd to Ru is determined to be 74.7 to 25.3, in consistent with the inductively coupled plasma optical emission spectrometry (ICP-OES) result (Supplementary Table 2). The powder X-ray diffraction (PXRD) pattern (Supplementary Fig. 5b) of Pd_3_Ru_1_ NPs shows five prominent diffraction peaks located at 39.8°, 46.2°, 67.5°, 81.3°, and 85.7°, assigned to the (111), (200), (220), (311), and (222) planes of *fcc* crystal phase, respectively, without observable shifts compared with those of commercial carbon-supported platinum nanoparticles (Pt/C), further confirming that Pd_3_Ru_1_ NPs have the same crystal structure and lattice parameters as Pt.

The deposition of atomic Pt overlayers on the obtained Pd_3_Ru_1_ NPs was performed in a layer-by-layer fashion through a wet-chemical approach (details in Methods). The well-defined Pd_3_Ru_1_/Pt_nL_ (n = 1–6) core/shell NPs could be achieved by controlling the amount of Pt precursor (Supplementary Fig. 6 and Table 3). The structural parameters of Pt shells were identified from the atomic stacking sequence and corresponding FFT pattern. The simulated high-resolution HAADF-STEM images of the surface atomic arrangement on the Pt shells with the corresponding FFT pattern are taken from the dashed squares in Fig. 1e, h, k, demonstrate that the corresponding Pt_nL_ shells are in accordance with the (111), (112), and (011)-oriented *fcc* structure, respectively. The atomic-resolution HAADF-STEM images (Fig. 1f, i) of one side view on the obtained NP reveals that the total thickness of NP increases to 8 atomic layers (counted by blue lines, Fig. 1f) and 12 atomic layers (counted by cyan lines, Fig. 1i), respectively. Compared with the original thickness of Pd_3_Ru_1_ NP with 6 atomic layers (Fig. 1b), the foreign Pt shell is determined to be about 1–2 monolayer thick in the Figs. 1f, and 3–4 monolayer thick in the Fig. 1i, respectively. Furthermore, a 6-atom-thick Pt shell (counted by red lines, Fig. 1k) was formed on the Pd_3_Ru_1_ NP with continuous lattice fringes across the interface, indicating the epitaxial growth of Pt overlayers on the Pd_3_Ru_1_ NPs. The EDS elemental mapping analyses further confirm the core/shell structures of Pd_3_Ru_1_/Pt_nL_ NPs (Fig. 1g, j, l), in which the countable atomic Pt shell and heterogeneous core can be distinguished from the contrast of different elemental signals. The integrated pixel intensities of the (111) lattices taken from various selected areas (boxed by the blue rectangular) of Pd_3_Ru_1_/Pt_1-2L_ NPs (Fig. 1f), Pd_3_Ru_1_/Pt_3-4L_ NPs (Fig. 1h) and Pd_3_Ru_1_/Pt_5-6L_ NPs (Fig. 1k) are shown in Fig. 1m, where all of the average (111) distances are calculated to be 2.26 Å, identical to Pd_3_Ru_1_ (111) (Fig. 1b) and bulk Pt (111) (Supplementary Fig. 4), illustrating inexistence of geometric strain in the primary {111} crystal planes of Pd_3_Ru_1_/Pt_1-6L_ NPs. The PXRD patterns (Fig. 1n) of various Pd_3_Ru_1_/Pt_1-6L_ NPs reveal that there are no shifts on five prominent diffraction peaks relative to those of Pd_3_Ru_1_ NPs and commercial Pt/C (Supplementary Fig. 5b), confirming the absence of strain in the Pt_1-6L_ shells. Furthermore, the geometric phase analysis (GPA) on the random Pd_3_Ru_1_/Pt_nL_ NPs was performed to recheck strain on the Pt shells. The corresponding false-colored GPA map shows almost uniform color throughout the NP, reconfirming a negligible in-plane strain (*ɛ*_xx_) on the Pt shells (Supplementary Fig. 7).

### Investigations of electronic structure and ligand effect

**Fig. 2:**
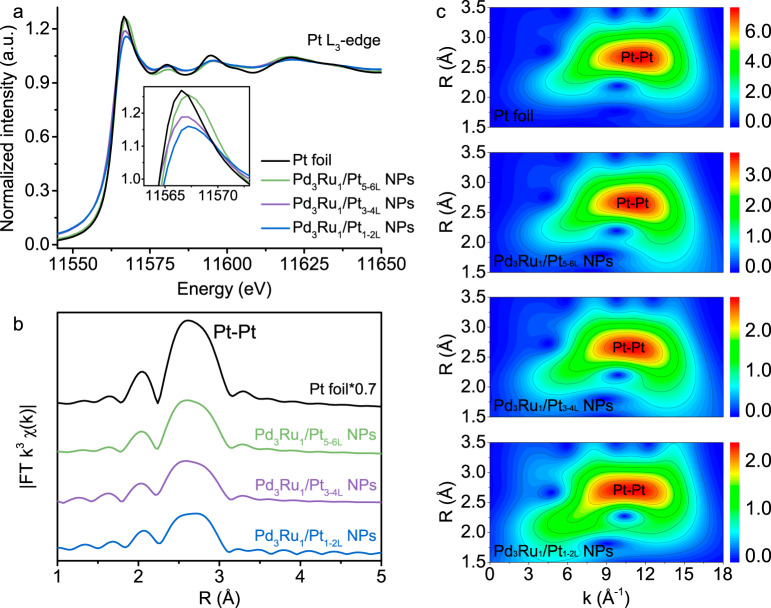
Spectroscopic characterization of Pd_3_Ru_1_/Pt_nL_ NPs and Pt foil. **a** XANES spectra at the Pt *L*_3_-edge. *Inset*, enlarged view of WL peak intensity. **b** *k*^3^-weighted FT-EXAFS spectra at *R* space. **c** Pt *L*_3_-edge WT analyses.

The electronic structure of Pt and the electronic interaction between Pd_3_Ru_1_ core and Pt shell were studied by X-ray absorption spectroscopy (XAS). Figure 2a displays the X-ray absorption near-edge structure (XANES) spectra of Pt *L*_3_-edge of various Pd_3_Ru_1_/Pt_nL_ NPs and Pt foil. The intensity of Pt *L*_3_-edge white line (WL) is regarded as a qualitative indicator of electron density in the 5*d* orbitals of Pt atoms^19^. As shown in Fig. 2a, the peak intensity of various catalysts in the Pt *L*_3_-edge WL region follows the order: Pt foil ˃ Pd_3_Ru_1_/Pt_5-6L_ NPs ˃ Pd_3_Ru_1_/Pt_3-4L_ NPs ˃ Pd_3_Ru_1_/Pt_1-2L_ NPs (*inset* of Fig. 2a), indicating that the electron density of Pt shell in the Pd_3_Ru_1_/Pt_nL_ NPs is successively increased as the thickness of Pt shell decreases.

Besides, the distances of Pt–Pt bonds were quantitatively investigated by Fourier transform extended X-ray absorption fine structure (FT-EXAFS) (Fig. 2b, Supplementary Fig. 8 and Table 4), and the corresponding atomic dispersions and bonding circumstances were displayed in the wavelet transform (WT) of *k* space data (Fig. 2c). It can be seen that the interatomic distance of Pt–Pt in the Pd_3_Ru_1_/Pt_5-6L_ NPs (2.75 Å), Pd_3_Ru_1_/Pt_3-4L_ NPs (2.75 Å) and Pd_3_Ru_1_/Pt_1-2L_ NPs (2.76 Å) is almost identical with that of Pt foil (2.76 Å), which rules out a geometric strain in the Pt shells of Pd_3_Ru_1_/Pt_nL_ NPs. In consideration of the excluded strain effect, the changed electron density of Pt in the Pd_3_Ru_1_/Pt_nL_ NPs can be assigned to the electronic effect that electron transfer induces the change of the *d*-band electron density of Pt^20^.

**Fig. 3:**
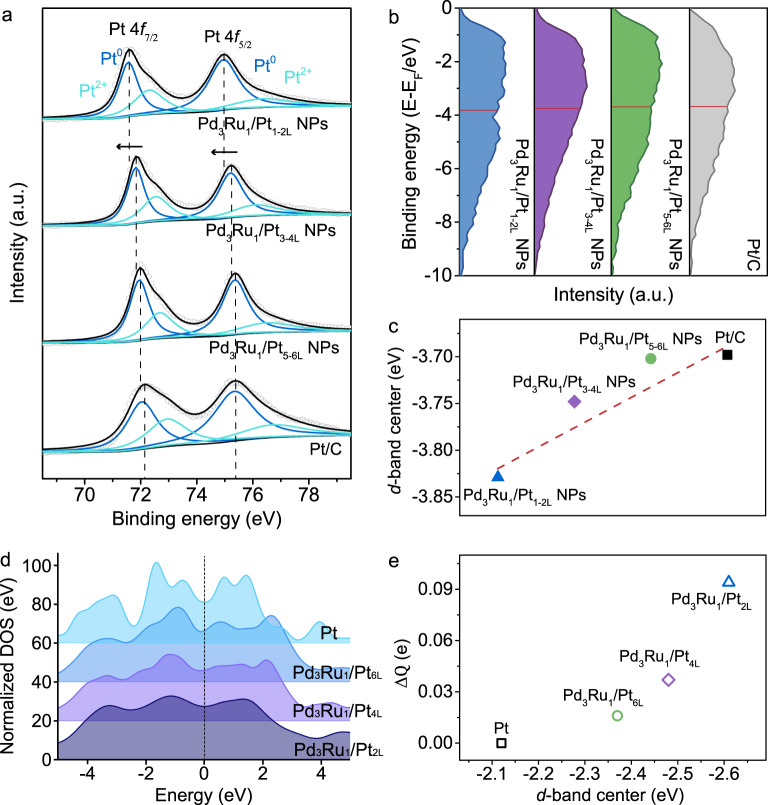
XPS and DFT analyses of electronic structure of surface Pt. **a** Pt 4*f* XPS spectra and **b** XPS valence band spectra measured for Pd_3_Ru_1_/Pt_1-2L_ NPs, Pd_3_Ru_1_/Pt_3-4L_ NPs, Pd_3_Ru_1_/Pt_5-6L_ NPs and commercial Pt/C. **c** The *d*-band center shifts calculated from the integration of XPS valence band spectra in **b**. **d** The normalized DOS and **e** Bader charge and DFT calculated *d*-band center of the Pd_3_Ru_1_/Pt_2L_ model, Pd_3_Ru_1_/Pt_4L_ model, Pd_3_Ru_1_/Pt_6L_ model, and pure Pt.

The changes in valence states of Pt shells and Pd_3_Ru_1_ core were studied by X-ray photoelectron spectroscopy (XPS). As displayed in Fig. 3a, the Pt 4*f* binding energy of Pt shells in the Pd_3_Ru_1_/Pt_nL_ NPs exhibits a gradient negative shift with a decreasing thickness of Pt shells whereas the Pd 3*d* and Ru 3*p* binding energies of Pd_3_Ru_1_/Pt_nL_ NPs show a gradient positive shift with the decreased Pt shells (Supplementary Fig. 9). The increased shifts of Pt 4*f*, Pd 3*d* and Ru 3*p* binding energies in the Pd_3_Ru_1_/Pt_nL_ NPs (from n: 5–6 L, to n: 3–4 L, to n: 1–2 L) represent an enhanced electronic effect that induced by electron transfer from Pd_3_Ru_1_ cores to Pt shells. According to the XPS valence-band spectra results (Fig. 3b), the *d*-band center of Pd_3_Ru_1_/Pt_nL_ NPs downshifts successively in the order of Pd_3_Ru_1_/Pt_5-6L_ NPs (−3.702 eV) > Pd_3_Ru_1_/Pt_3-4L_ NPs (−3.748 eV) > Pd_3_Ru_1_/Pt_1-2L_ NPs (−3.829 eV), revealing a shift of *d*-band center of Pt_nL_ away from the Fermi level in comparison with Pt/C (−3.698 eV) (Fig. 3c). Therefore, the electronic structure of surface Pt is tuned by the electronic effect from Pd_3_Ru_1_ core, leading to a downshift of Pt *d*-band center with respect to the Fermi level. Such electronic interaction between Pt shells and heterogenous cores (ligand effect) in the Pd_3_Ru_1_/Pt_1-6L_ configuration is much stronger in the case of monolayer Pt (Pt_1-2L_) and becomes weak (Pt_3-4L_) or even disappears (Pt_5-6L_) with increasing the thickness of Pt shell.

DFT calculation was performed to give a theoretical analysis of the *d*-band center shift and Bader charge of Pt. As shown in the Fig. 3d, the peak of normalized *d*-projected density of states (DOS) of Pt surfaces in different models (Supplementary Fig. 10) demonstrates a smoothing trend from pristine Pt, to Pt_6L_, to Pt_4L_ then to Pt_2L_, indicating that bonding states between atoms become weaker in the presence of thinner Pt layers of Pd_3_Ru_1_/Pt_nL_ model. The weaker bond interactions, the greater the deviation of the local average of the *d* electron energies of Pt_nL_ from the benchmark value of pristine Pt. Hence, the *d*-band center of respective Pt_nL_ shows up reasonable downshift relative to that of pure Pt (-2.12 eV) away from the Fermi level, as the order of Pd_3_Ru_1_/Pt_6L_ (−2.37 eV) > Pd_3_Ru_1_/Pt_4L_ (−2.48 eV) > Pd_3_Ru_1_/Pt_2L_ (−2.61 eV) (Fig. 3e), which is consistent with the experimental results.

The Bader charge (Δ*Q*) analysis of Pt_nL_ was further carried out to investigate the average electron transfer between Pt_nL_ and Pd_3_Ru_1_, and the pure Pt without charge transfer (Δ*Q* = 0) was utilized as a reference. As displayed in the Fig. 3e, the Pd_3_Ru_1_/Pt_2L_ showcases the greatest number of electrons being transferred; approximately 0.097e contributes from Pd_3_Ru_1_ to Pt_2L_. Likewise, the Pd_3_Ru_1_/Pt_4L_ and the Pd_3_Ru_1_/Pt_6L_ exhibit weaker charge transfers of 0.037e and 0.016e from Pd_3_Ru_1_ to Pt_4L_ and Pt_6L_, respectively. This result theoretically confirms that effective charge transfer at the interface are pronounced in the Pd_3_Ru_1_/Pt_2L_; meanwhile, the thicker of Pt_nL_, the weaker the electronic effect in the Pd_3_Ru_1_/Pt_nL_.

### Electrocatalytic ORR

**Fig. 4:**
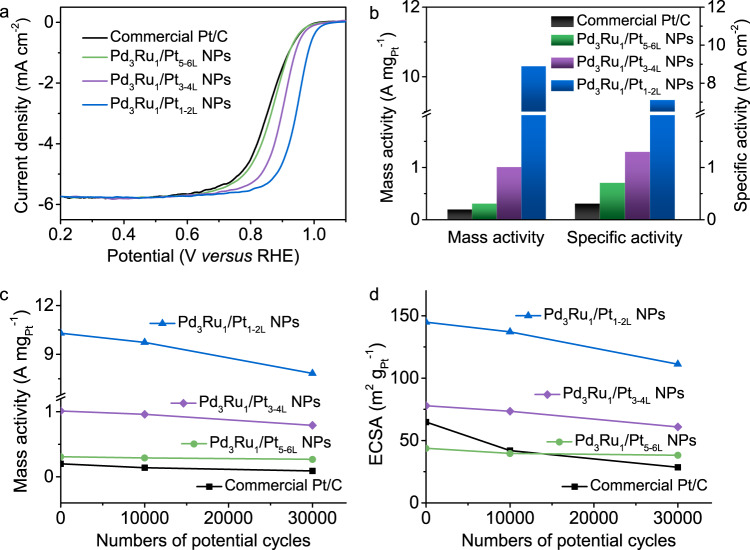
Electrocatalytic performance of Pd_3_Ru_1_/Pt_nL_ NPs and commercial Pt/C catalysts for ORR. **a** ORR polarization curves were recorded in O_2_-saturated 0.1 M KOH solution at a sweep rate of 20 mV/s and a rotation rate of 1600 rpm. **b** Column diagrams of MA and SA of different catalysts at 0.9 V *versus* RHE. **c** MA changes and **d** ECSA changes of different catalysts before and after 10,000 and 30,000 potential cycles between 0.6 V and 1.0 V *versus* RHE.

The ligand effect in the Pd_3_Ru_1_/Pt_nL_ NPs catalysts on ORR activity was examined in the O_2_-saturated 0.1 M KOH solution. The commercial Pt/C (20.0 wt% of 3 nm-Pt nanoparticles supported on carbon) was used as a benchmark catalyst (Supplementary Fig. 11). The ORR polarization curves of different catalysts at a rotation rate of 1600 rpm and a sweep rate of 20 mV/s at room temperature were recorded in Fig. 4a. The Pd_3_Ru_1_/Pt_1-2L_ NPs catalyst exhibits the highest half-wave potential (*E*_1/2_) of 0.945 V, higher than those of Pd_3_Ru_1_/Pt_3-4L_ NPs (0.900 V), Pd_3_Ru_1_/Pt_5-6L_ NPs (0.867 V) and commercial Pt/C (0.855 V), suggestive of its most prominent activity toward ORR. Normalized with Pt loadings, the MAs of different catalysts at 0.9 V were calculated to follow by the order of Pd_3_Ru_1_/Pt_1-2L_ NPs (10.3 A/mg_Pt_) > Pd_3_Ru_1_/Pt_3-4L_ NPs (1.01 A/mg_Pt_) > Pd_3_Ru_1_/Pt_5-6L_ NPs (0.31 A/mg_Pt_) > commercial Pt/C (0.20 A/mg_Pt_) (Fig. 4b) whereas normalized with the electrochemical active surface areas (Supplementary Table 5), the specific activities (SA) follow the order of Pd_3_Ru_1_/Pt_1-2L_ NPs (7.11 mA/cm^2^) > Pd_3_Ru_1_/Pt_3-4L_ NPs (1.30 mA/cm^2^) > Pd_3_Ru_1_/Pt_5-6L_ NPs (0.71 mA/cm^2^) > commercial Pt/C (0.31 mA/cm^2^). As is known, an ideal ORR electrocatalyst should have a weaker binding energy to oxygen relative to Pt (111) by 0.2 eV, and an optimal downshift in the *d*-band center should be 0.2 eV lower than that of Pt^9^. In agreement with the *d*-band model, the Pd_3_Ru_1_/Pt_1-2L_ NPs, with a downshift in the *d*-band center of 0.131 eV lower than that of Pt (the nearest to optimal value), show the highest ORR activity among different Pd_3_Ru_1_/Pt_nL_ NPs catalysts, and also are better than most of the reported Pt-based catalysts (Supplementary Table 6).

We also find that the Pd_3_Ru_1_/Pt_1-2L_ NPs with strong ligand effect show the remarkable electrocatalytic MA of 10.3 A/mg_Pt_ and SA of 7.11 mA/cm^2^, 51.5-fold and 22.9-fold higher than those of commercial Pt/C catalysts, whereas the Pd_3_Ru_1_/Pt_3-4L_ NPs with the weak ligand effect that triggered a slight downshift in the *d*-band center show 5.05-fold and 4.19-fold higher MA and SA than those of commercial Pt/C catalysts, respectively, and the Pd_3_Ru_1_/Pt_5-6L_ NPs catalyst shows the comparable ORR activity as that of commercial Pt/C. The above results further reveal that a downshift of the *d*-band center of Pt induced by the ligand effect enhances the ORR catalytic activity.

The ORR electrochemical durability tests were measured in O_2_-saturated 0.1 M KOH solution by sweeping the potential cycles between 0.6 and 1.0 V at a scanning rate of 50 mV/s (Supplementary Fig. 12). The changes of MAs at 0.9 V and electrochemically active surface areas (ECSAs) during the tests were summarized in Fig. 4c and d. After 30,000 potential cycles, the MA of the Pd_3_Ru_1_/Pt_1-2L_ NPs was still as high as 7.83 A/mg_Pt_, 39.2-fold higher than that of the initial commercial Pt/C catalyst, and 87.0-fold higher than that of commercial Pt/C catalyst over 30,000 potential cycles. Furthermore, the Pd_3_Ru_1_/Pt_3-4L_ NPs and Pd_3_Ru_1_/Pt_5-6L_ NPs retain 78.2% and 87.1% of the initial MA after 30,000 potential cycles, respectively, much higher than that of commercial Pt/C catalyst (45.0%) (Fig. 4c). As for ECSAs over 30,000 potential cycles (Fig. 4d), there remain high for the Pd_3_Ru_1_/Pt_nL_ NPs with Pt_1-2L_, Pt_3-4L_, and Pt_5-6L_ by maintaining 76.7 %, 78.2%, and 87.2% of their initial values, respectively, superior to Pt/C catalyst with the maintenance of 44.1%. Furthermore, the Pd_3_Ru_1_/Pt_nL_ NPs show negligible change in their hexagonal morphology (Supplementary Fig. 13) after 30,000 potential cycles; however, the commercial Pt/C catalyst displays severe nanoparticulate aggregation under the same test condition (Supplementary Fig. 14). The high ORR durability of Pd_3_Ru_1_/Pt_nL_ NPs is believed to be relevant to the electronic interaction between Pd_3_Ru_1_ and Pt that renders surface Pt atoms less oxophilic and more antioxidative during the catalytic process^16,21,22^.

The ligand effect of the Pd_3_Ru_1_/Pt_nL_ NPs catalysts in ORR activity was further examined in the O_2_-saturated 0.1 M HClO_4_ solution (Supplementary Fig. 15a). The MAs of different catalysts at 0.9 V follow the order of Pd_3_Ru_1_/Pt_1-2L_ NPs (4.59 A/mg_Pt_) > Pd_3_Ru_1_/Pt_3-4L_ NPs (0.93 A/mg_Pt_) > Pd_3_Ru_1_/Pt_5-6L_ NPs (0.38 A/mg_Pt_) > commercial Pt/C (0.18 A/mg_Pt_), whereas their SAs show the same order of Pd_3_Ru_1_/Pt_1-2L_ NPs (3.16 mA/cm^2^) > Pd_3_Ru_1_/Pt_3-4L_ NPs (1.19 mA/cm^2^) > Pd_3_Ru_1_/Pt_5-6L_ NPs (0.87 mA/cm^2^) > commercial Pt/C (0.28 mA/cm^2^) (Supplementary Fig. 15b). The variation trend for ORR activities of Pd_3_Ru_1_/Pt_nL_ NPs catalysts with exclusive ligand effect in the acidic electrolyte is consistent with the results under alkaline conditions. The optimal Pd_3_Ru_1_/Pt_1-2L_ NPs also render a competitive ORR activity in acid electrolytes among reported state-of-the-art Pt-based core/shell catalysts (Supplementary Table 7). The durability tests (Supplementary Fig. 16) reveal that the Pd_3_Ru_1_/Pt_nL_ NPs retain more than half of the initial values of MAs and ECSAs after 30,000 potential cycles, respectively (n = 1–2 L, 52.1% and 52.8%; n = 3–4 L, 58.1% and 59.7%; n = 5–6 L, 57.9% and 67.1%), higher than those of commercial Pt/C catalyst (27.8% and 38.4%) (Supplementary Fig. 17a, b). Worth mentioning that the ORR activity of Pd_3_Ru_1_ NPs was also examined in alkaline and acidic electrolytes, respectively (Supplementary Fig. 18). The Pd_3_Ru_1_ NPs are inert toward ORR, excluding the physical effect of Pd_3_Ru_1_ to the improvement of ORR catalytic performance.

### Correlation of ORR activities and *d*-band centers under exclusive ligand effect

Sabatier principle has guided the advancing of heterogeneous catalysis for almost one century and well maps the activity of catalytic elements (especially transition metals) as a function of binding energy in a volcano manner^23^. Each time when the catalytic element of interest (Pt in the case of ORR discussed here) is not satisfactorily efficient, it is customary to introduce another element to appropriately shift the catalytic element toward the peak *via* the so-called alloying strategy. Furthermore, configurating such alloys in various core-shell manners has proven benefits to the ORR electrocatalysis in terms of specific and mass activities, as well as stabilities. This improvement has been generally ascribed to the co-presence of ligand and strain effects that tune the oxygen adsorption toward the optimum. To clarify the origin, it is crucial to quantify how much each effect contributes to the activity improvement. One decade ago, Strasser and co-workers reported the first attempt to separate the strain effect from the convolution by using a dealloyed catalyst model^24^. With that model, a relation between ORR activity and the lattice strain of catalytic Pt could be experimentally established. However, to date, there is no reported experimental effort to separate and quantitate ligand effect in a real catalytic structure, although this effect is widely assumed to play a major role in promoting electrocatalysis over alloys. This study aims to fill this knowledge gap.

**Fig. 5:**
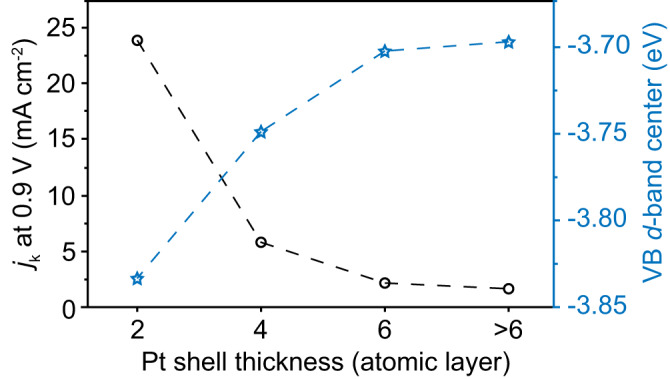
Correlation of ORR activities and *d*-band centers of Pt shell with different thickness under exclusive ligand effect. Differences of kinetic current density (*j*_k_ at 0.9 V) and *d*-band center calculated from valence-band spectra, respectively, between Pd_3_Ru_1_/Pt_nL_ and commercial Pt/C catalysts.

Experimentally, we avoided the generation of surface strain by designing an alloy composition (Pd_3_Ru_1_) with the same lattice parameter as pure Pt. Our presented microscopic and spectroscopic evidence (Figs. 1 and 2) strongly supported the key assumption of our work that the as-constructed core/shell structure (Pd_3_Ru_1_/Pt_nL_) generated no surface strain. On the other hand, studies on the electronic structures of Pd_3_Ru_1_/Pt_nL_ by XPS and DFT (Fig. 3) confirmed the shift of the *d*-band center of Pt *via* the exclusive ligand effect. It was also evident that the extent of such exclusive ligand effect can be experimentally tuned by controlling the thickness of the Pt shell, which then motivated us to map ORR activities and *d*-band centers as a function of the number of atomic layers of the Pt shell, as shown in Fig. 5. We stress here that Fig. 5 is unprecedented because all involved parameters are experimentally measurable, and their variations are due to exclusive ligand effect. Most of the prior structure-performance relations were given based on either computational or ligand-and-strain convoluted electronic structure parameters. For the first time, our strategy enables the experimental visualization of exclusive ligand effect in heterogeneous catalysis and is expected to provoke future efforts to re-examine the role of ligand effect (experimentally) in other electrocatalytic reactions relevant to renewable energy conversions.

## Discussion

In conclusion, we design and precisely synthesize a class of Pd_3_Ru_1_/Pt_nL_ core/shell model catalyst with exclusive ligand effect for greatly boosting the electrocatalytic activity. We experimentally and theoretically demonstrate that the electron transfer from the Pd_3_Ru_1_ core to Pt can result in an increase of electron density in the 5*d* orbitals of Pt, thus the downshift of the *d*-band center of Pt, which can lead to greatly enhanced catalytic performance. The ligand-effect optimized Pd_3_Ru_1_/Pt_1-2L_ NPs show extraordinary half-wave potential of 0.945 V and MA of 10.3 A/mg_Pt_ at 0.9 V *versus* reversible hydrogen electrode (*vs*. RHE) towards ORR in alkaline electrolyte, which is among the best ORR Pt-based catalysts. In acid electrolytes, the Pd_3_Ru_1_/Pt_1-2L_ NPs also show a superior MA of 4.59 A/mg_Pt_ for ORR. They are stable for ORR in both alkaline/acid electrolytes by showing MA decay of 21.8%/47.9% after 30,000 potential cycles, less than those of commercial Pt/C catalysts (55.0%/72.2%). The present work provides the first experimental evidence for exclusive ligand effect on electrocatalysis and also reports a promising class of core/shell nanostructured electrocatalysts for fuel cells bottlenecked ORR.

## Methods

### Synthesis of Pd_*x*_Ru_1−*x*_ NPs

In a typical synthesis of Pd_*x*_Ru_1−*x*_ NPs, the designated amounts of Pd(acac)_2_, Ru_3_(CO)_12_, and ascorbic acid were added to 5.0 mL oleylamine in a 15 mL high-pressure glass vial (Chongqing Synthware Glass, P160001). Then, the glass vial was sealed, and the mixture was stirred in an oil bath at 45 °C for 1 h to get a homogeneous solution. The solution was heated to 180 °C for 6 h until cooling to room temperature. The resulting black colloidal product was collected by centrifugation (at a speed of 8000*g* for 5 min), washed three times with cyclohexane and ethanol, and redispersed in 10 mL oleylamine for further use.

The amounts of corresponding reagents in the synthesis of the Pd_97_Ru_3_ NPs, Pd_85_Ru_15_ NPs, Pd_75_Ru_25_ NPs, and Pd_50_Ru_50_ NPs were summarized in Supplementary Table 1, respectively.

### Synthesis of Pd_3_Ru_1_/Pt_nL_ NPs

Firstly, 3.3 mL Pd_3_Ru_1_ NPs oleylamine solution was dispersed in 15 mL benzyl alcohol and ultrasonicated for 30 min. Simultaneously, a Pt precursor solution was prepared by dissolving Pt(acac)_2_ in the mixture of oleylamine and oleic acid. The as-prepared 18.3 mL solution of Pd_3_Ru_1_ NPs was injected into a 100 mL flask and heated at 180 °C with stirring for 15 min. Then, the Pt precursor solution was injected into the reaction flask at a rate of 5.00 mL h^−1^. After the injection, the mixed solution was heated at 180 °C for 1 h with stirring. Subsequently, the reaction mixture was cooled down to room temperature and added to 50.0 mL of cyclohexane/ethanol (volume ratio: 2:1). Finally, the Pd_3_Ru_1_/Pt_nL_ NPs were collected by centrifugation (at a speed of 8000*g* for 5 min) and washed twice with a mixture of cyclohexane/ethanol.

The amounts of Pt(acac)_2_, oleylamine, and oleic acid in the synthesis of the Pd_3_Ru_1_/Pt_1-2L_ NPs, Pd_3_Ru_1_/Pt_3-4L_ NPs, and Pd_3_Ru_1_/Pt_5-6L_ NPs were summarized in Supplementary Table 3, respectively.

### Characterization

TEM was conducted on HITACHI HT7700 at an accelerating voltage of 100 kV. High-angle annular dark-field scanning TEM (HAADF-STEM) and energy dispersive X-ray spectroscopy (EDS) element mappings were done on an aberration-corrected JEOL 2200FS STEM/TEM microscope at 300 kV equipped with a Bruker-AXS SDD detector. X-ray diffraction (XRD) patterns were collected on the Rigaku D/max-2500 powder diffractometer with Cu-Kα radiation (*λ* = 0.15406 nm). The specific elemental composition of Pd_x_Ru_1-x_ NPs and Pd_3_Ru_1_/Pt_nL_ NPs and the concentration of catalysts were determined by an inductively coupled plasma optical emission spectrometer (Agilent 5110 ICP-OES). The X-ray absorption fine-structure spectroscopy (XAFS) at Pt *L*_3_-edge was performed at the 1W1B beamline of Beijing Synchrotron Radiation Facility (BSRF, 2.5 GeV, a maximum current of 250 mA, Si (311) double-crystal). All samples were tableted and measured at room temperature under fluorescence mode. The Athena module of the IFEFFIT software package was employed to analyze the acquired XAFS raw data according to the standard data analysis procedures, and the data fitting was performed using the Artemis in IFEFFIT^25^. The X-ray photoelectron spectroscopy (XPS) was conducted with the Thermo Scientific K-AlphaK spectrometer. Based on the XPS valence band spectra, the *d*-band center of gravity (${\varepsilon }_{d}$) of the metal catalyst was calculated in the range of −10.0 to 0 eV by using the following Eq. (1).

1 $${\varepsilon }_{d}=\frac{\int N\left(\varepsilon \right)\varepsilon d\varepsilon }{\int N\left(\varepsilon \right)d\varepsilon }$$

### Electrochemical measurements

The Pd_3_Ru_1_/Pt_nL_ NPs catalysts were prepared by depositing the as-synthesized Pd_3_Ru_1_/Pt_1-2L_ NPs, Pd_3_Ru_1_/Pt_3-4L_ NPs and Pd_3_Ru_1_/Pt_5-6L_ NPs onto the carbon black (Vulcan XC-72), respectively. In a typical preparation, the Pd_3_Ru_1_/Pt_nL_ NPs dispersed in cyclohexane were mixed with carbon black dispersed in ethanol under ambient sonication for 1 h. After stirring for another 6 h, the product was collected by centrifugation at the speed of 8000*g* for 5 min, and washed twice with ethanol, then dried at 70 °C for 3 h. Before the electrochemical tests, we did further thermal treatment of the obtained catalysts at 220 °C for 2 h in an N_2_ atmosphere to clean up the organic residue on the nanocrystals. The metal (Pd + Ru + Pt) loading amount on carbon was controlled to be about 20.0 wt%, and the actual loading was analyzed by ICP-OES.

The catalyst inks with a concentration of 1.00 mg_catalysts_/mL were prepared by dispersing the catalyst powder into a mixture of isopropanol, ultrapure water, and Nafion solution with a volume ratio of 1:1:0.0025 through sonication. The specific Pt loadings of commercial Pt/C ink, Pd_3_Ru_1_/Pt_5-6L_ NPs ink, Pd_3_Ru_1_/Pt_3-4L_ NPs ink, and Pd_3_Ru_1_/Pt_1-2L_ NPs ink were 0.200, 0.147, 0.111, and 0.045 mg_Pt_/mL, respectively. Then, 10 μL of catalyst inks was dropped on the surface of working electrodes for the electrochemical tests.

All electrochemical tests were carried out in a three-electrode system by using a rotating disk electrode device (Pine Research Instrumentation, USA) connected to a CHI750e electrochemical workstation (Shanghai Chenhua Instrument Corporation, China) at room temperature. In detail, a glassy carbon rotating disk electrode with a diameter of 5.0 mm was used as the working electrode, Pt foil (1.0 × 1.5 cm^2^) was used as a counter electrode, and Hg/HgO electrode or saturated calomel electrode was used as a reference electrode for alkaline or acid electrolyte, respectively. The electrochemically active surface area (ECSA) of the catalyst was determined by integrating the hydrogen adsorption charge on the cyclic voltammetry (CV) with a scan rate of 50 mV/s at room temperature in N_2_-saturated 0.1 M HClO_4_ solution. To evaluate the ORR kinetics of the above-mentioned catalysts, the linear scan voltammetry (LSV) measurements were performed in O_2_-saturated 0.1 M KOH/HClO_4_ solution at a scan rate of 20 mV/s with a rotating rate of 1600 r/min. Besides, the ORR electrochemical durability tests were measured in O_2_-saturated 0.1 M KOH/HClO_4_ solution by conducting CV curves at the potential between 0.6 and 1.0 V *versus* RHE at a sweep rate of 50 mV/s for 10,000 and 30,000 potential cycles, respectively.

### DFT calculations

Our total energy calculations were performed within the framework of DFT using the projector augmented plane-wave (PAW) method, as implemented in the Vienna ab initio Simulation Package (VASP)^26^. The generalized gradient approximation (GGA) proposed by Perdew, Burke, and Ernzerhof (PBE) was selected for the exchange-correlation potential^27^. A long-range van der Waals interaction was described by the DFT-D3 approach^28^. The cut-off energy for the expanding plane wave was set to 450 eV. The energy criterion was treated as 10–5 eV in the iterative solution of the Kohn–Sham equation. We built three configurations of the surface superstructure with respect to experimental observations: n-layer (n = 2, 4, 6) (2 × 2) Pt shell on Pd_3_Ru_1_ surface. The 2-layer pristine Pt was also built as a reference. To avoid artificial interaction between periodic images, a vacuum layer of 16 Å was added along the perpendicular z direction of the crystalline structure. The Brillouin zone integration was sampled using a Monkhorst–Pack (3 × 3 × 1) k-mesh. The atomic positions were fully relaxed until the residual forces on the per atom were less than 0.01 eV/Å. For DOS simulation, a (19 × 19 × 1) k-mesh was used. The normalized DOS was utilized to analyze the electron contributions in the Pd_3_Ru_1_/Pt_nL_ configurations and the normalized DOS was defined as the total DOS of Pd_3_Ru_1_/Pt_nL_ divided by the number of Pt layers, such as 2 layers, 4 layers, and 6 layers. The average charge transfer was determined by the following Eq. (2).

2 $$\Delta {{Q}}=\frac{1}{{{n}}({{Q_{{{{{\rm{a}}}}}}}}-{{Q_{{{{{\rm{b}}}}}}}})}$$

Δ*Q* represents the difference in the Bader charge, *Q*_a_ and *Q*_b_ described the atomic Bader charge of Pd_3_Ru_1_/Pt_nL_ and original states of the valence electron in pristine Pt, n was the number of Pt atoms in the corresponding Pd_3_Ru_1_/Pt_nL_ model.

### Supplementary information

Supplementary Information

Peer Review File

### Source data

Source Data

## Data Availability

The data generated in this study are available within the paper and Supplementary Information. Source data are provided in this paper.
