# Supplementary material for: Precise synthetic control of exclusive ligand effect boosts oxygen reduction catalysis
Source: Nat Commun. 2023 Oct 28;14:6893. doi: 10.1038/s41467-023-42514-w (PMC10613207; doi:10.1038/s41467-023-42514-w)
Supplement: Supplementary file 1 — Supplementary Information [file 41467_2023_42514_MOESM1_ESM.pdf]

Supplementary information

Precise synthetic control of exclusive ligand effect boosts oxygen reduction catalysis

Lu Tao[1,2,4], Kai Wang[1,4], Fan Lv[1], Hongtian Mi[2], Fangxu Lin[1], Heng Luo[1], Hongyu Guo[1], Qinghua Zhang[2], Lin Gu[2], Mingchuan Luo[1] & Shaojun Guo[1]*

[1]School of Materials Science and Engineering, Peking University, Beijing, 100871, China.

[2]School of Materials Science and Engineering, University of Science and Technology Beijing, Beijing, 100083, China.

[3]Beijing National Laboratory for Condensed Matter and Institute of Physics, Chinese Academy of Sciences, Beijing, 100190, China.

[4]These authors contributed equally to this work.

*Corresponding author: E-mail: guosj@pku.edu.cn

Chemicals. Dodecacarbonyl triruthenium ($Ru_3(CO)_{12}$, 99%) was bought from Acros. Palladium diacetylacetonate ($Pd(acac)_2$), 99.9%), platinum diacetylacetonate ($Pt(acac)_2$), 99%), oleylamine (OAm, 70%), oleic acid (OA, 90%), perchloric acid ($HClO_4$, 70%) and Pd/C catalyst (10 wt%) were obtained from Sigma-Aldrich. L-ascorbic acid (L-AA, 99%), isopropanol, ethanol and cyclohexane were all supplied by Beijing Tongguang Fine Chemicals Company. Benzyl alcohol, potassium hydroxide (KOH, 95%) and Ru/C (5 wt%) were purchased from Aladdin. Commercial JM Pt/C catalyst (20 wt%, 3±1 nm Pt nanoparticles supported on carbon black) was bought from Johnson Matthey Catalysts, and Nafion D-521 dispersion (5 wt%) was obtained from Alfa Aesar. All the chemicals were used as received without further purification. The deionized water (18.2 MΩ cm^{-1}) used in all experiments was prepared by passing through an ultrapure purification system.

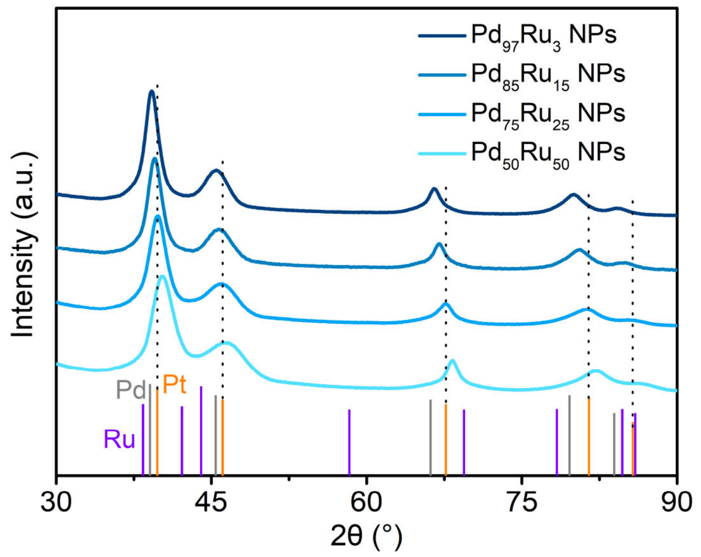

Supplementary Fig. 1 PXRD patterns of different Pd_xRu_{1-x} NPs. PDF cards: Pt 04-0802; Pd 87-0637; Ru 06-0663.

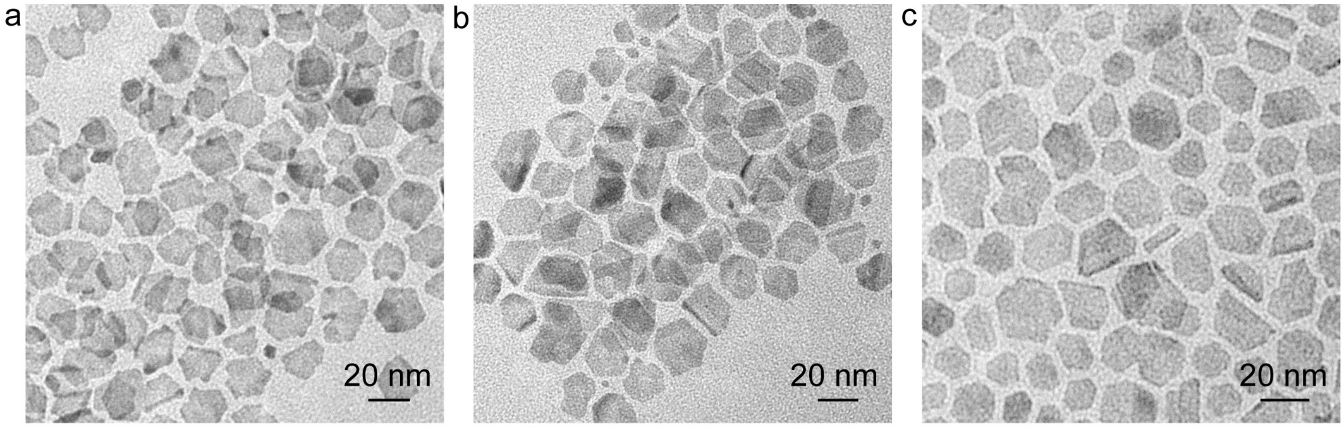

Supplementary Fig. 2 TEM images of (a) $Pd_{97}Ru_3$ NPs, (b) $Pd_{85}Ru_{15}$ NPs and (c) $Pd_{50}Ru_{50}$ NPs.

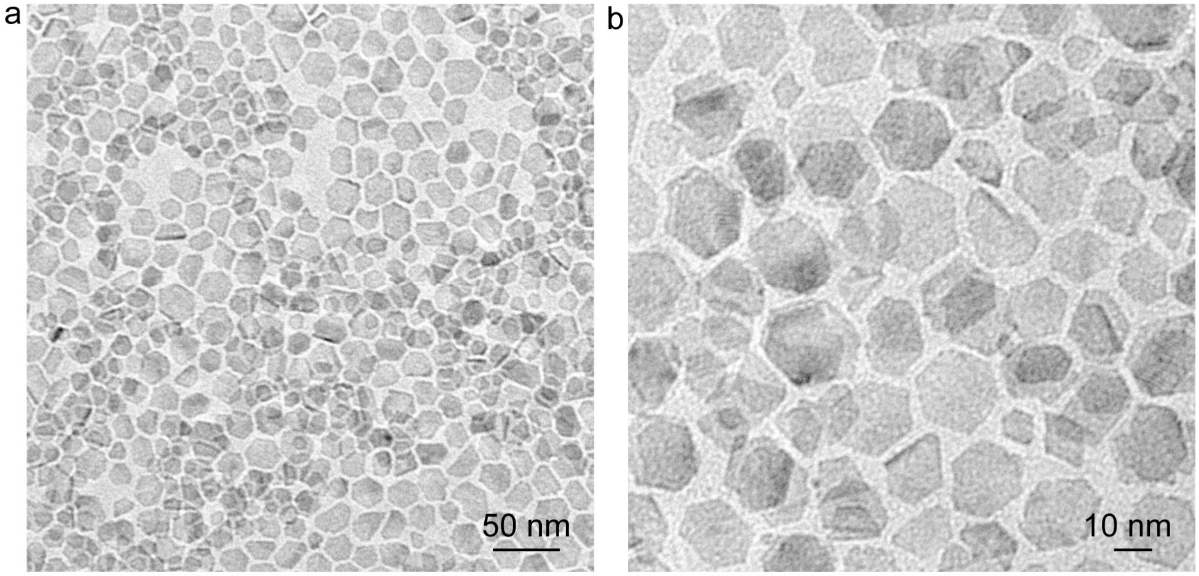

Supplementary Fig. 3 Low-magnification (a) and high-magnification (b) TEM images of Pd_3Ru_1 NPs.

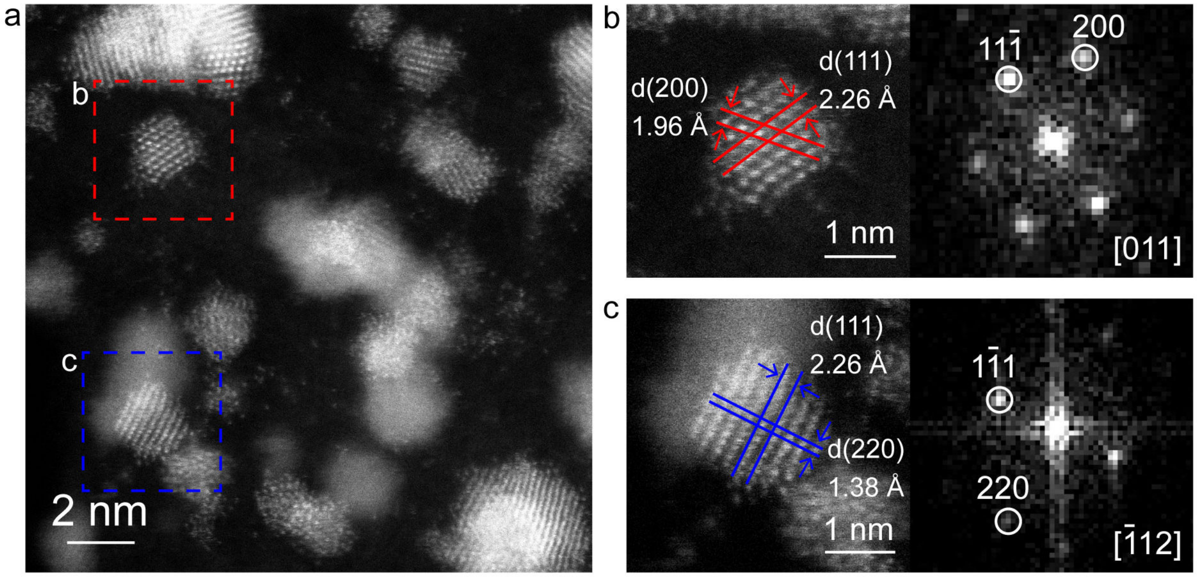

Supplementary Fig. 4 (a) HAADF-STEM image of commercial Pt/C. (b, c) High-resolution HAADF-STEM images of atomic arrangement on Pt with the corresponding FFT pattern taken from the red and blue square in (a), respectively.

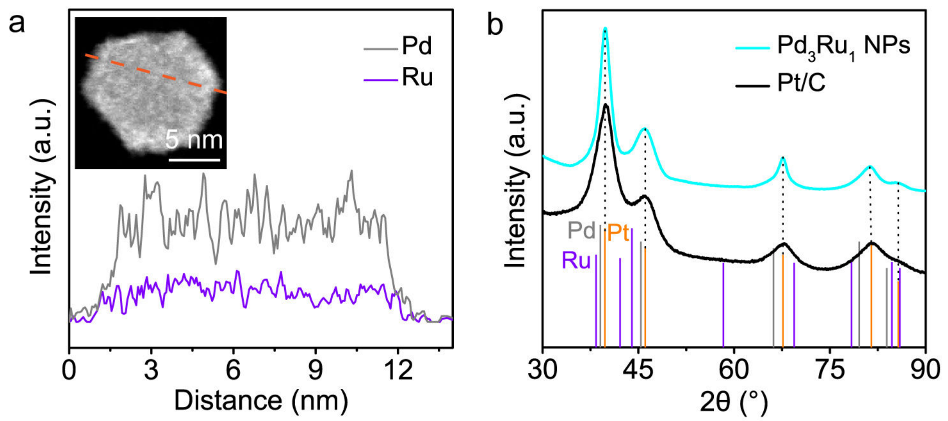

Supplementary Fig. 5 (a) EDS line-scan profile across the Pd_3Ru_1 NP marked by the orange dashed line in the inset. (b) PXRD patterns of Pd_3Ru_1 NPs and commercial Pt/C. PDF card: Pd 87-0637, Ru 06-0663, Pt 04-0802.

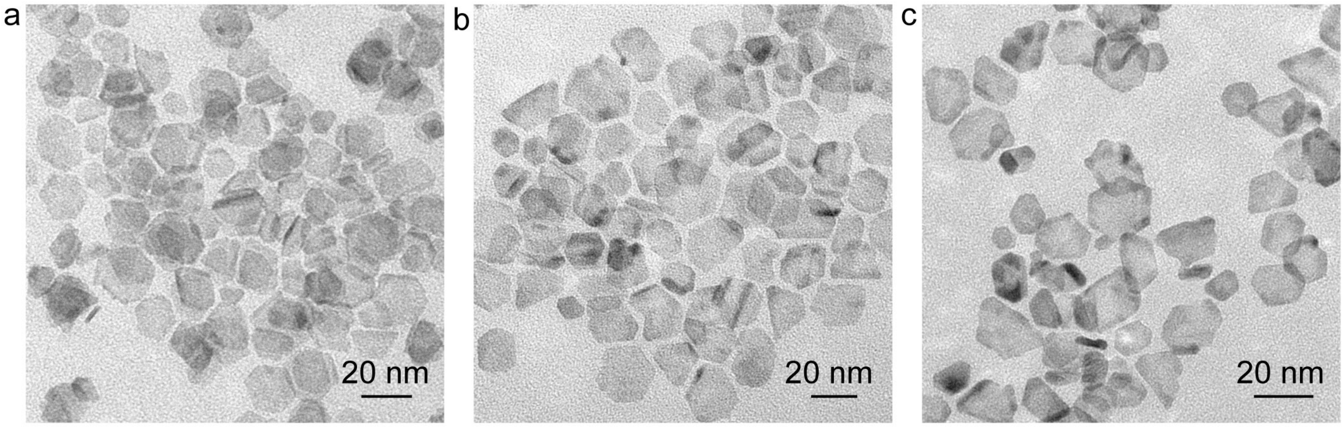

Supplementary Fig. 6 TEM images of (a) $Pd_3Ru_1/Pt_{1\text{-}2L}$ NPs, (b) $Pd_3Ru_1/Pt_{3\text{-}4L}$ NPs and (c) $Pd_3Ru_1/Pt_{5\text{-}6L}$ NPs.

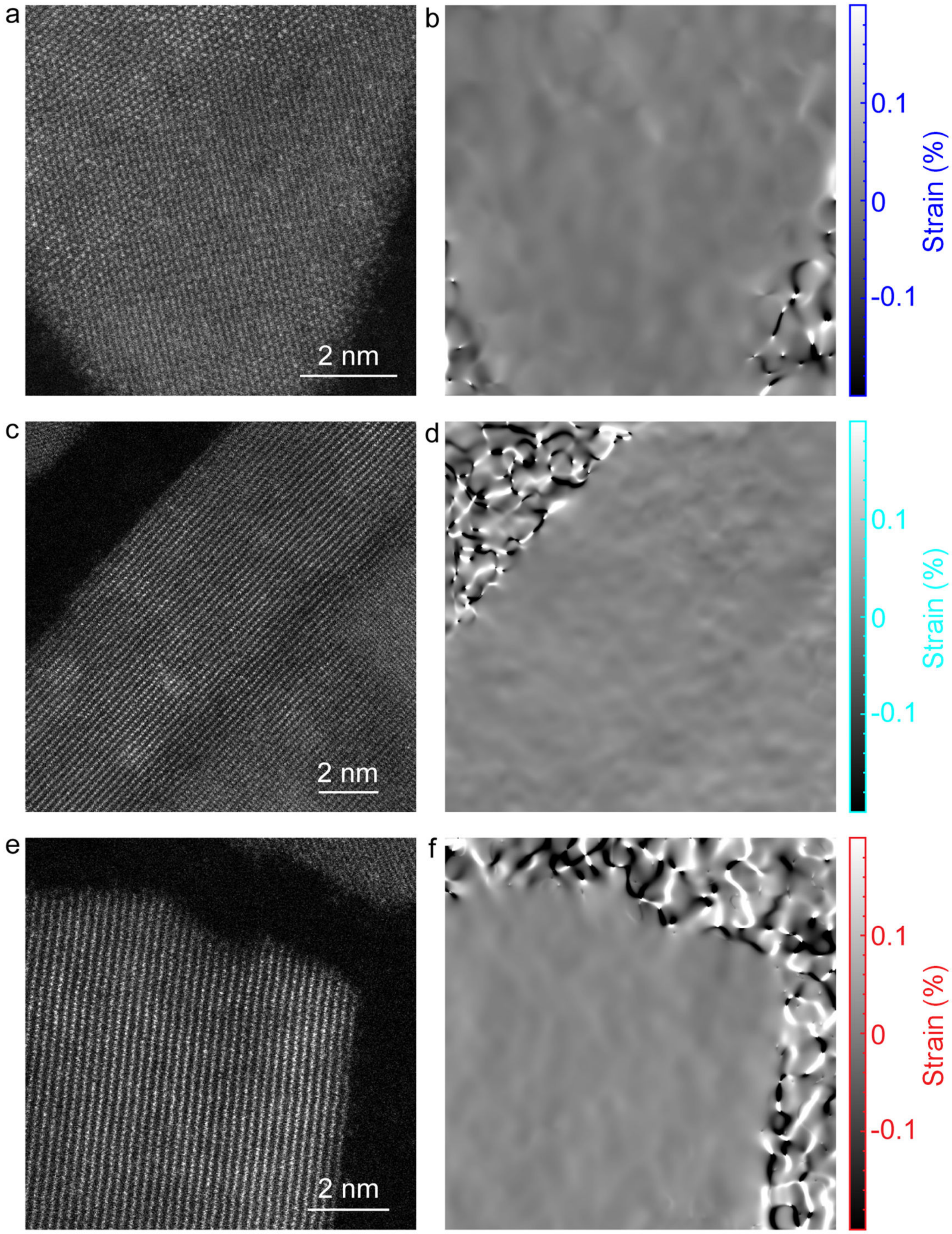

Supplementary Fig. 7 (a, c, e) Atomic-resolution HAADF-STEM images and (b, d, f) corresponding false-colored GPA maps of in-plane strain (ε_{xx}) field of (a, b) $Pd_3Ru_1/Pt_{1\text{-}2L}$ NP, (c, d) $Pd_3Ru_1/Pt_{3\text{-}4L}$ NP and (e, f) $Pd_3Ru_1/Pt_{5\text{-}6L}$ NP.

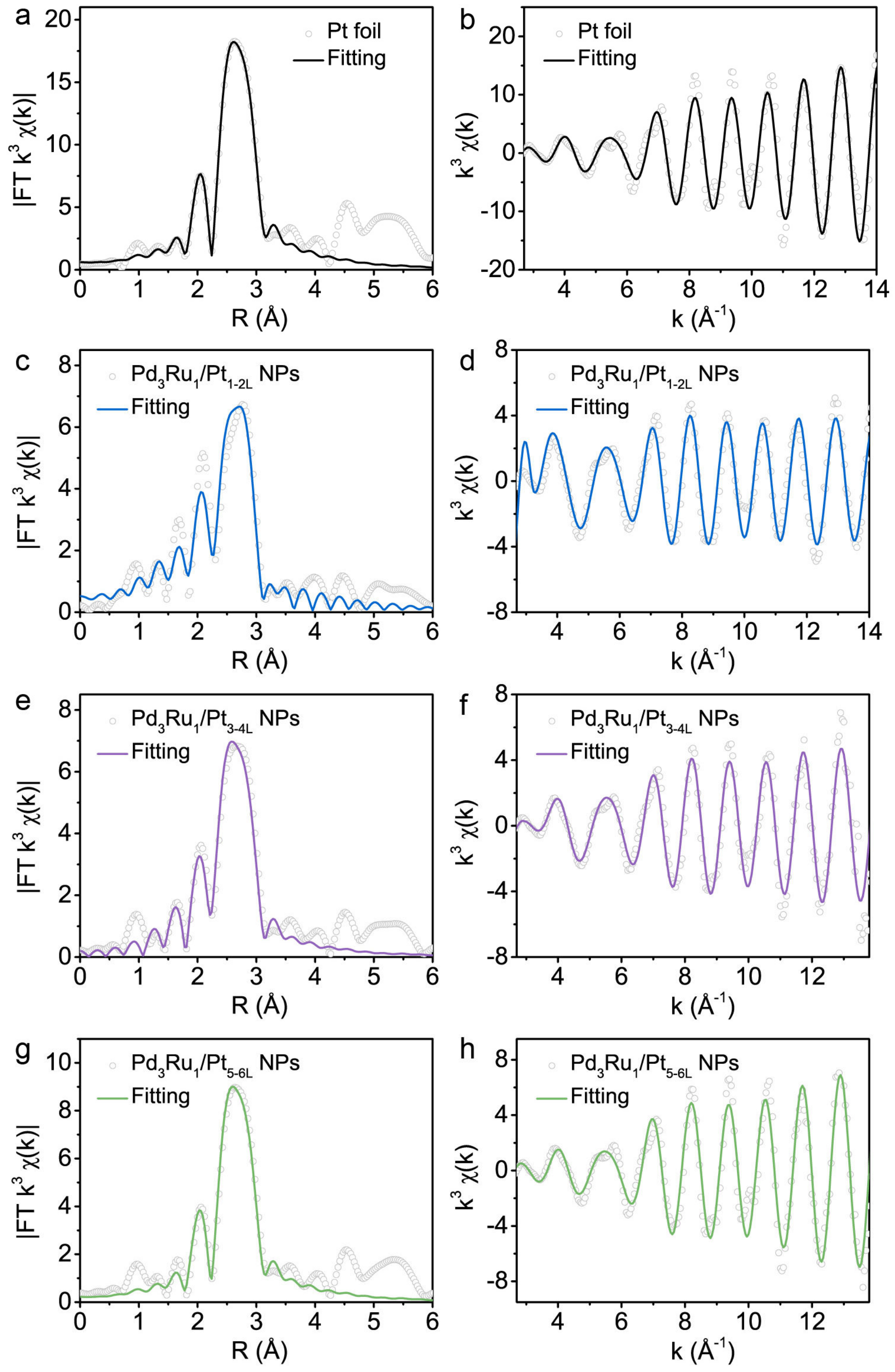

Supplementary Fig. 8 The *R* space and *k* space fitting results of Pt L_3-edge of (a, b) Pt foil, (c, d) $Pd_3Ru_1/Pt_{1\text{-}2L}$ NPs, (e, f) $Pd_3Ru_1/Pt_{3\text{-}4L}$ NPs and (g, h) $Pd_3Ru_1/Pt_{5\text{-}6L}$ NPs, respectively.

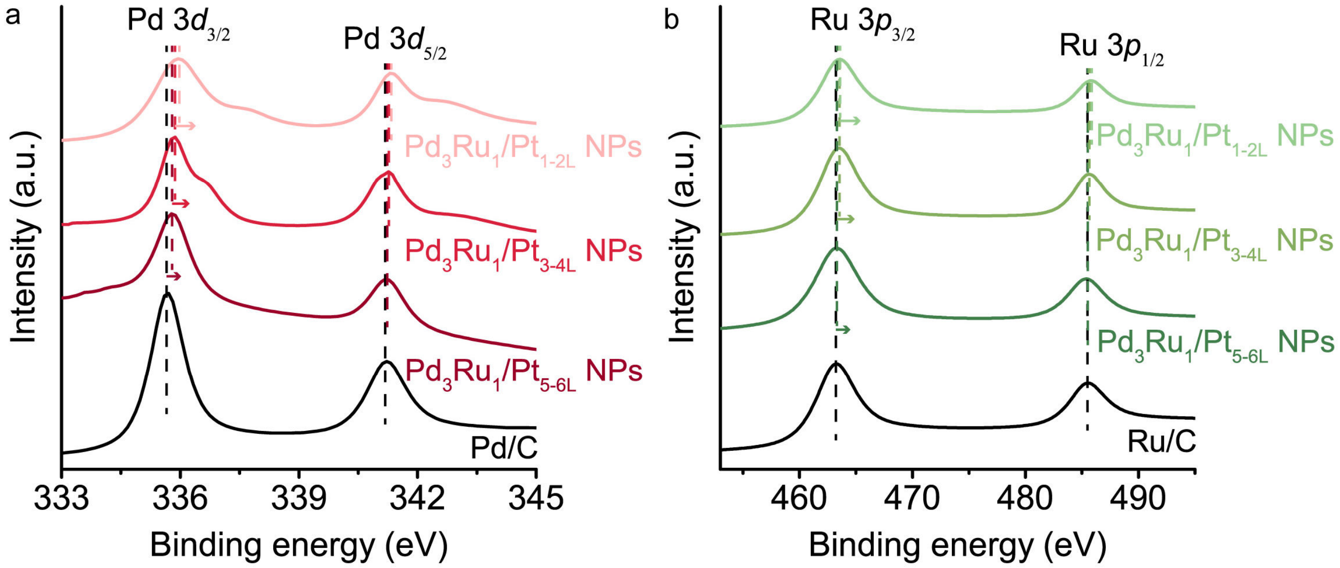

Supplementary Fig. 9 (a) Pd 3*d* XPS spectra of Pd_3Ru_1/Pt_{nL} NPs and Pd/C. (b) Ru 3*p* XPS spectra of Pd_3Ru_1/Pt_{nL} NPs and Ru/C.

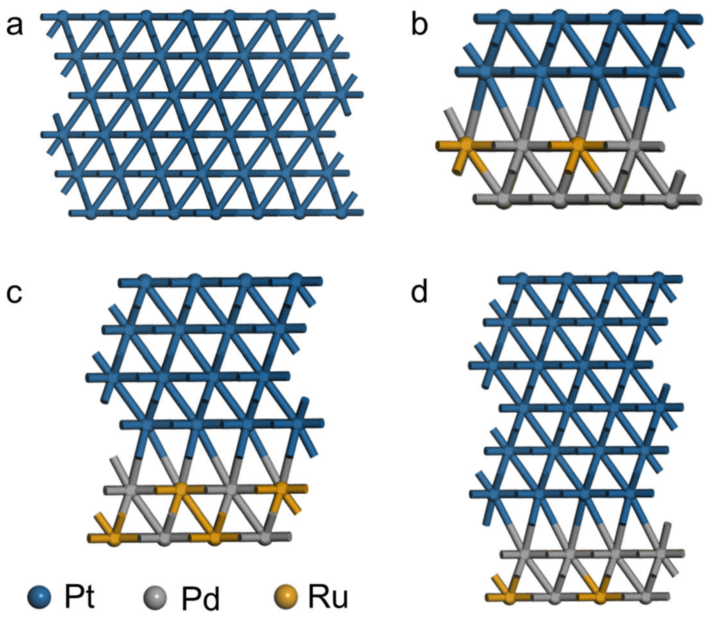

Supplementary Fig. 10 The structure models of (a) Pt, (b) $Pd_3Ru_1/Pt_{1\text{-}2L}$, (c) $Pd_3Ru_1/Pt_{3\text{-}4L}$ and (d) $Pd_3Ru_1/Pt_{5\text{-}6L}$.

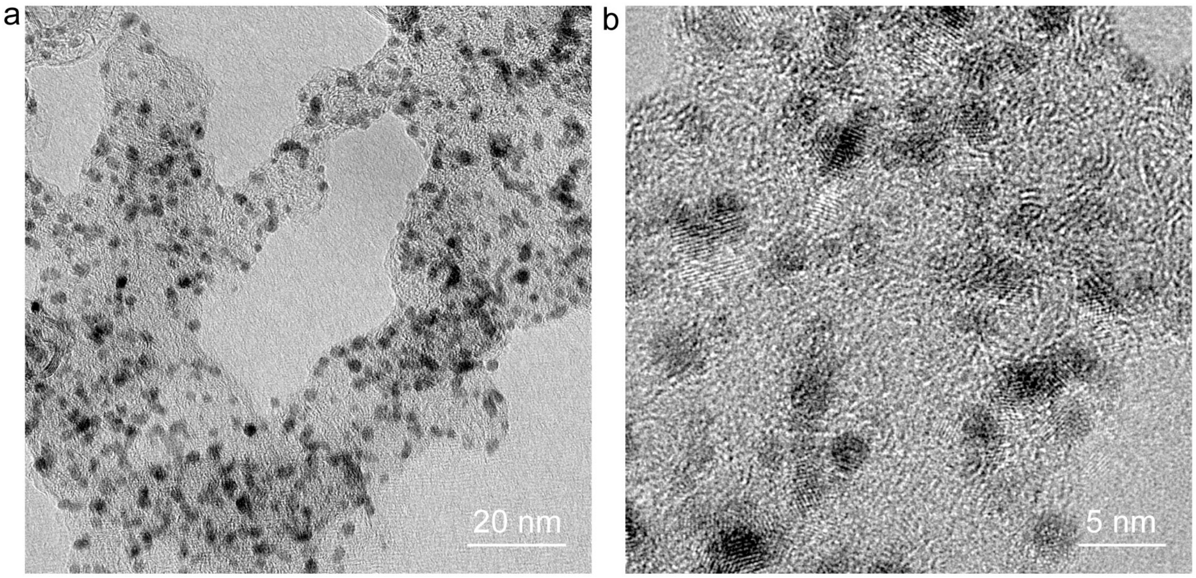

Supplementary Fig. 11 (a) Low-magnificationand (b) high-magnification TEM images of commercial Pt/C catalyst.

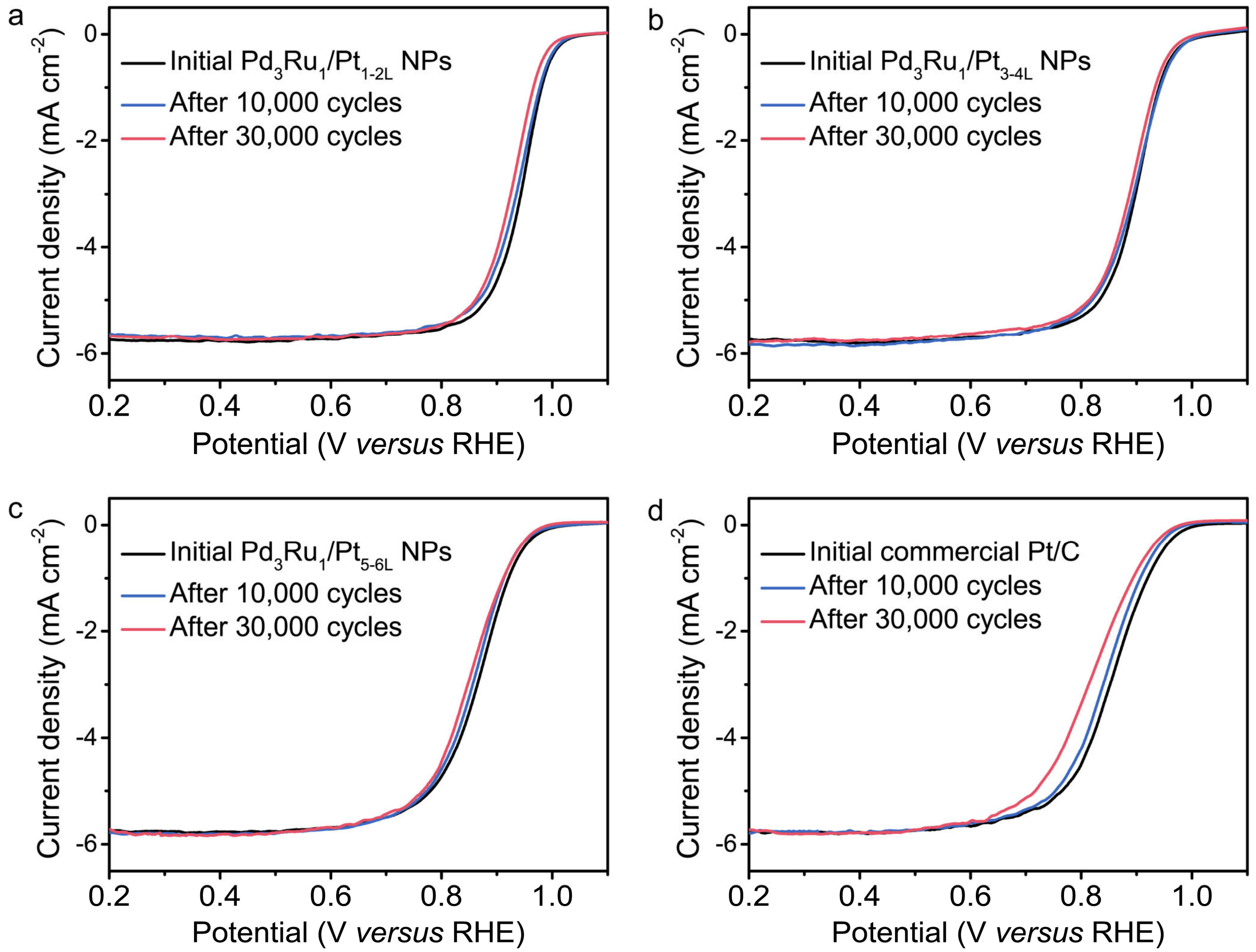

Supplementary Fig. 12 (a-d) ORR polarization curves of different catalysts in O_2-saturated 0.1 M KOH solution at a sweep rate of 20 mV/s and a rotation rate of 1,600 rpm before and after the durability tests.

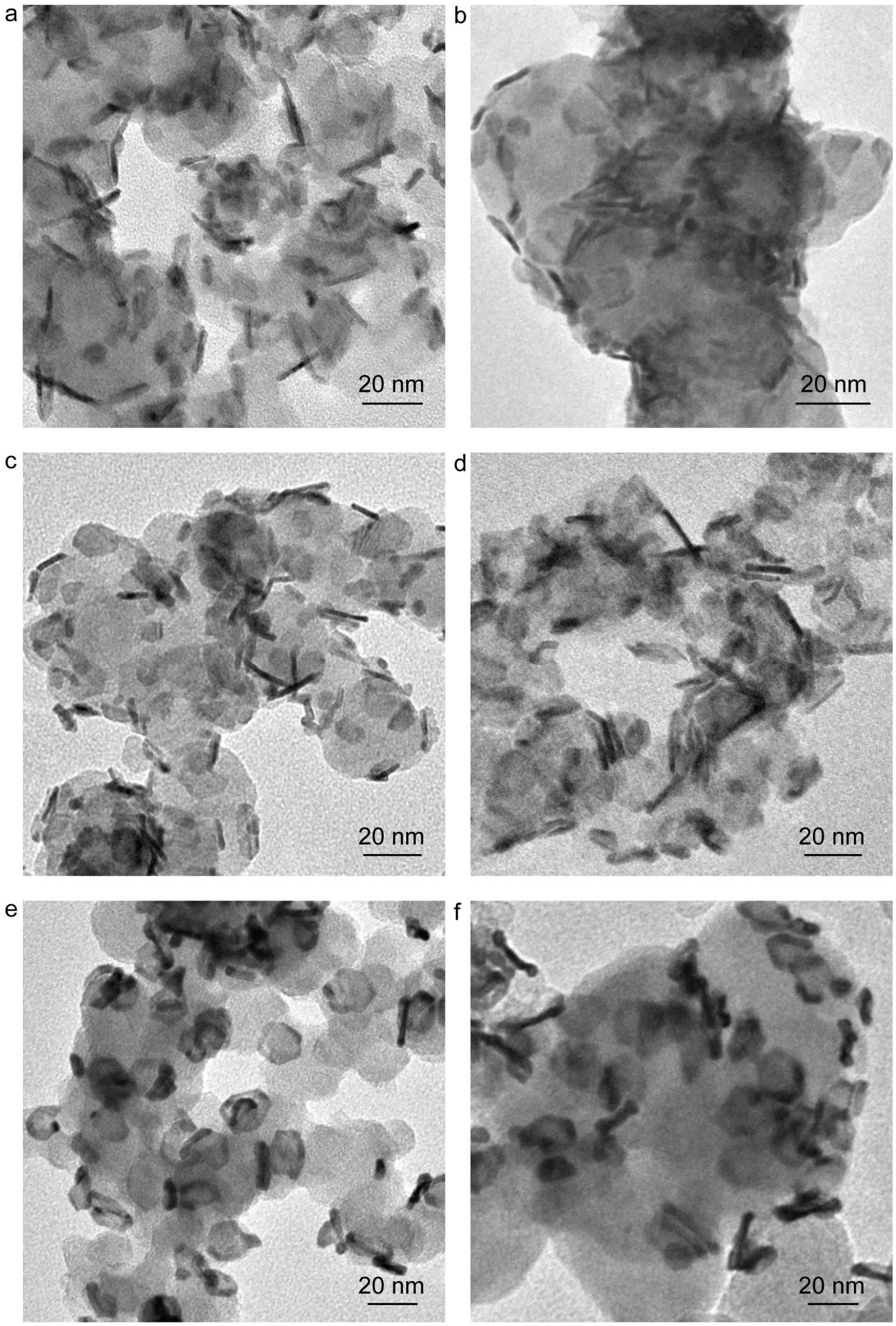

Supplementary Fig. 13 TEM images of $Pd_3Ru_1/Pt_{1\text{-}2L}$ NPs/C before (a) and after (b) 30,000 potential cycles in alkaline electrolyte. TEM images of $Pd_3Ru_1/Pt_{3\text{-}4L}$ NPs/C before (c) and after (d) 30,000 potential cycles in alkaline electrolyte. TEM images of $Pd_3Ru_1/Pt_{5\text{-}6L}$ NPs/C before (e) and after (f) 30,000 potential cycles in alkaline electrolyte.

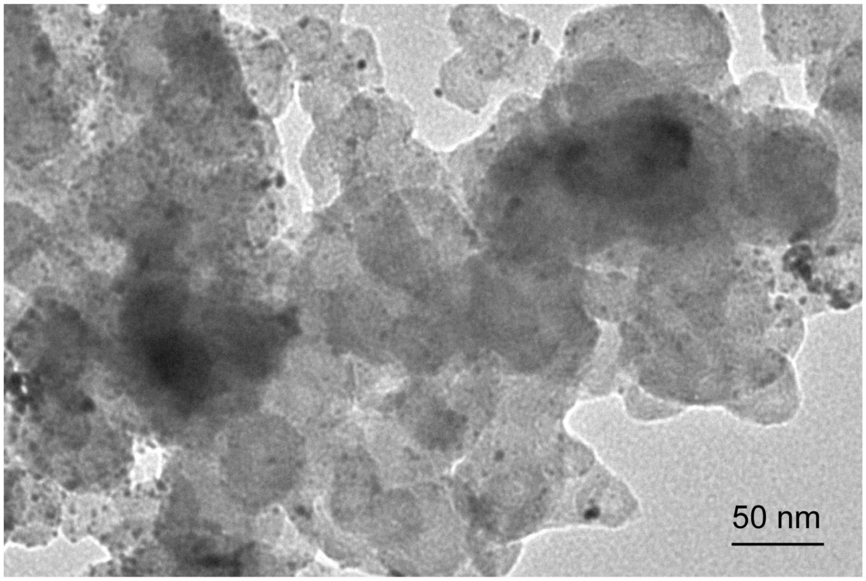

Supplementary Fig. 14 TEM image of commercial Pt/C catalyst after 30,000 potential cycles in alkaline electrolyte.

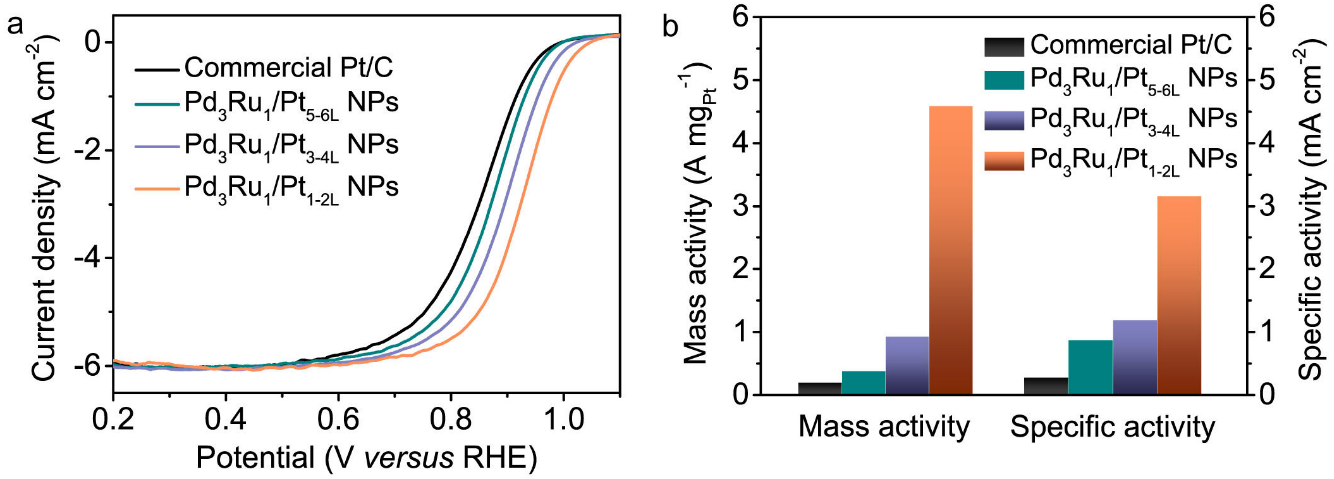

Supplementary Fig. 15 Electrocatalytic performances of Pd_3Ru_1/Pt_{nL} NPs and commercial Pt/C catalysts for ORR in the acidic electrolyte. (a) ORR polarization curves were recorded in O_2-saturated 0.1 M $HClO_4$ solution at a sweep rate of 20 mV/s and a rotation rate of 1,600 rpm. (b) The MA and SA of different catalysts at 0.9 V *versus* RHE.

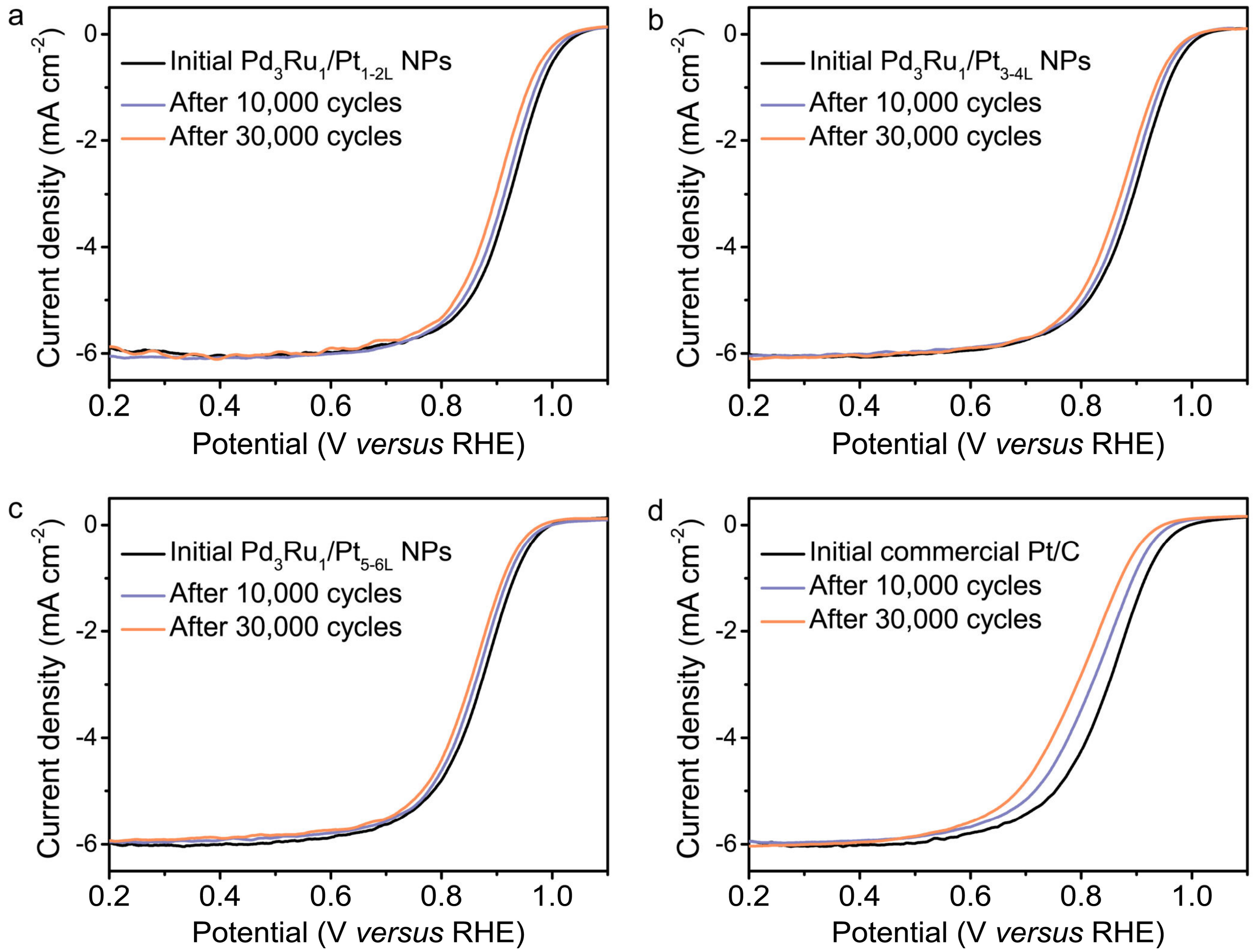

Supplementary Fig. 16 (a-d) ORR polarization curves of different catalysts in O_2-saturated 0.1 M $HClO_4$ electrolyte at a sweep rate of 20 mV/s and a rotation rate of 1,600 rpm before and after the durability tests.

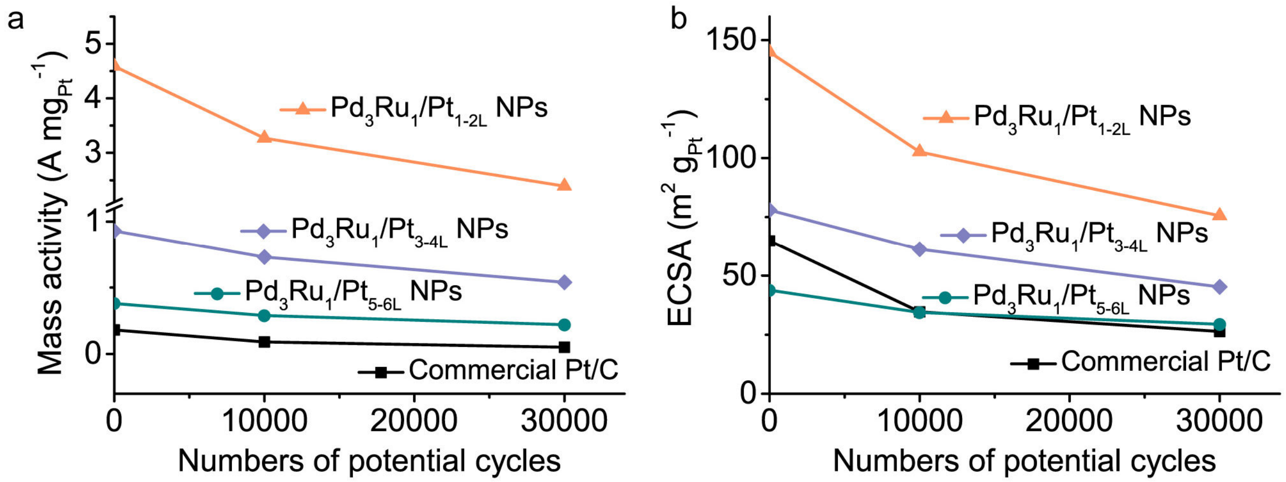

Supplementary Fig. 17 (a) MA changes and (b) ECSA changes of different catalysts before and after 10,000 and 30,000 potential cycles between 0.6 V and 1.0 V *versus* RHE in O_2-saturated 0.1 M $HClO_4$ electrolyte.

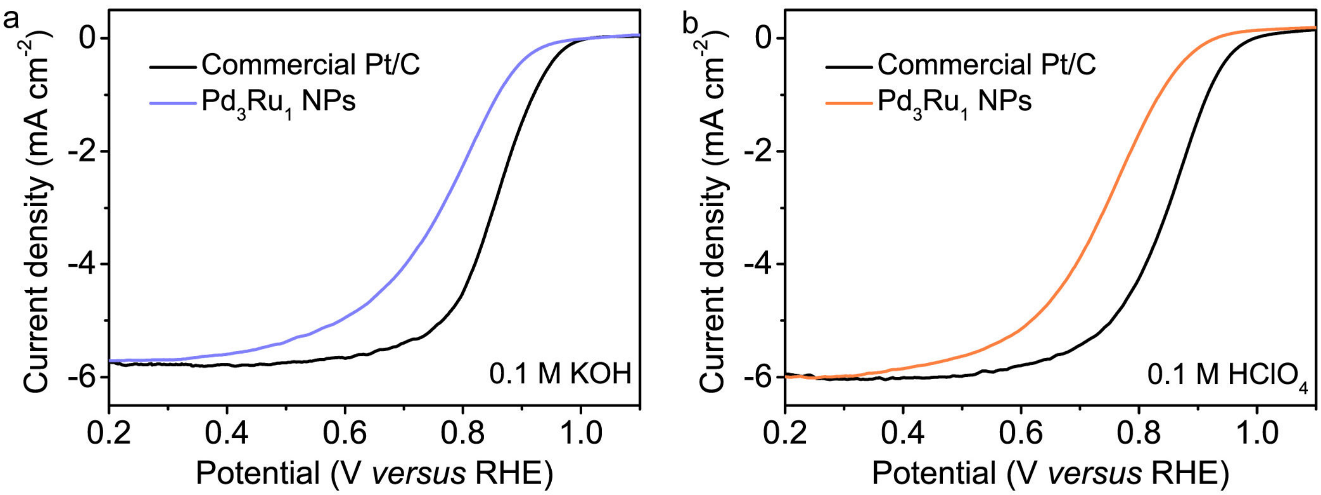

Supplementary Fig. 18 ORR polarization curves of Pd_3Ru_1 NPs and commercial Pt/C in O_2-saturated (a) 0.1 M KOH solution and (b) 0.1 M $HClO_4$ solution at a sweep rate of 20 mV/s and a rotation rate of 1,600 rpm.

Supplementary Table 1. The amount of chemicals used in the fabrication of Pd_xRu_{1-x} NPs.

Chemical agents / Samples	$Pd(acac)_2$ (mg)	$Ru_3(CO)_{12}$ (mg)	AA (mg)
$Pd_{97}Ru_3$ NPs	274.2	16.0	40.0
$Pd_{85}Ru_{15}$ NPs	274.2	32.0	40.0
$Pd_{75}Ru_{25}$ NPs (referred to as Pd_3Ru_1 NPs)	137.1	32.0	40.0
$Pd_{50}Ru_{50}$ NPs	45.7	32.0	40.0

Supplementary Table 2. ICP-OES results of different catalysts.

Samples \ Metals	Pd (wt%)	Ru (wt%)	Pt (wt%)
$Pd_{97}Ru_3$ NPs	96.9	0.31	-
$Pd_{85}Ru_{15}$ NPs	85.8	14.2	-
$Pd_{75}Ru_{25}$ NPs (referred to as Pd_3Ru_1 NPs)	75.2	24.8	-
$Pd_{50}Ru_{50}$ NPs	53.1	46.9	-
$Pd_3Ru_1/Pt_{1\text{-}2L}$ NPs	58.0	19.5	22.5
$Pd_3Ru_1/Pt_{3\text{-}4L}$ NPs	33.5	11.0	55.5
$Pd_3Ru_1/Pt_{5\text{-}6L}$ NPs	19.5	7.00	73.5

Supplementary Table 3. The amount of chemicals used in the fabrication of Pd_3Ru_1/Pt_{nL} NPs.

Products \ Chemical agents	$Pt(acac)_2$ (mg)	Oleylamine (mL)	Oleic acid (mL)
Pd_3Ru_1/Pt_{1-2L} NPs	5.00	2.00	2.00
Pd_3Ru_1/Pt_{3-4L} NPs	10.0	3.50	3.50
Pd_3Ru_1/Pt_{5-6L} NPs	25.0	5.00	5.00

Supplementary Table 4. EXAFS fit parameters at the Pt L_3-edge for various samples.

Sample	Shell	CN^a	R (Å)^b	σ^2 (Å^2)^c	ΔE_0 (eV)^d	R-factor
Pt foil	Pt-Pt	12.0	2.76±0.01	0.0045±0.0003	7.80±0.59	0.01
Pd_3Ru_1/Pt_{1-2L} NPs	Pt-Pt	5.5±1.6	2.76±0.02	0.0069±0.0014	9.49±2.93	0.03
Pd_3Ru_1/Pt_{3-4L} NPs	Pt-Pt	6.4±0.6	2.75±0.02	0.0060±0.0006	7.75±0.97	0.01
Pd_3Ru_1/Pt_{5-6L} NPs	Pt-Pt	6.6±0.6	2.75±0.02	0.0050±0.0005	7.59±0.72	0.01

- k range: 0.3-1.3 nm^{-1}.
- R range: 0.14-0.33 nm^{-1}.
- *a*: Coordination numbers.
- *b*: Bond length.
- *c*: Debye-Waller factors.
- *d*: Inner potential correction.
- *R* factor: Goodness of fit.
- S_0^2 was set to 0.82, according to the experimental EXAFS fit of Pt foil reference.

Supplementary Table 5. The electrochemical active surface areas of the catalysts.

Catalysts	ECSAs (m^2/g_{Pt})
$Pd_3Ru_1/Pt_{1\text{-}2L}$ NPs/C	144.9
$Pd_3Ru_1/Pt_{3\text{-}4L}$ NPs/C	77.9
$Pd_3Ru_1/Pt_{5\text{-}6L}$ NPs/C	43.8
Commercial Pt/C	64.8
$Pd_3Ru_1/Pt_{1\text{-}2L}$ NPs/C after 10,000 potential cycles (KOH)	137.1
$Pd_3Ru_1/Pt_{3\text{-}4L}$ NPs/C after 10,000 potential cycles (KOH)	73.5
$Pd_3Ru_1/Pt_{5\text{-}6L}$ NPs/C after 10,000 potential cycles (KOH)	39.7
Commercial Pt/C after 10,000 potential cycles (KOH)	41.9
$Pd_3Ru_1/Pt_{1\text{-}2L}$ NPs/C after 30,000 potential cycles (KOH)	111.2
$Pd_3Ru_1/Pt_{3\text{-}4L}$ NPs/C after 30,000 potential cycles (KOH)	60.9
$Pd_3Ru_1/Pt_{5\text{-}6L}$ NPs/C after 30,000 potential cycles (KOH)	38.2
Commercial Pt/C after 30,000 potential cycles (KOH)	28.6
$Pd_3Ru_1/Pt_{1\text{-}2L}$ NPs/C after 10,000 potential cycles ($HClO_4$)	102.6
$Pd_3Ru_1/Pt_{3\text{-}4L}$ NPs/C after 10,000 potential cycles ($HClO_4$)	61.2
$Pd_3Ru_1/Pt_{5\text{-}6L}$ NPs/C after 10,000 potential cycles ($HClO_4$)	34.4
Commercial Pt/C after 10,000 potential cycles ($HClO_4$)	34.7
$Pd_3Ru_1/Pt_{1\text{-}2L}$ NPs/C after 30,000 potential cycles ($HClO_4$)	77.9
$Pd_3Ru_1/Pt_{3\text{-}4L}$ NPs/C after 30,000 potential cycles ($HClO_4$)	46.5
$Pd_3Ru_1/Pt_{5\text{-}6L}$ NPs/C after 30,000 potential cycles ($HClO_4$)	29.4
Commercial Pt/C after 30,000 potential cycles ($HClO_4$)	25.2

Supplementary Table 6. Pt-based catalysts towards ORR in 0.1 M KOH electrolyte.

Catalysts	Morphology	Half-wave potential (V)	MA (A/mg_{Pt})	SA (mA/cm^2)	Reference
Pd_3Ru_1/Pt_{1-2L} NPs	PdRu/Pt core/shell nanoplates	0.945	10.3	7.11	This work
Pt_1Ni_3 NPs	Pt_1Ni_3 nanoparticles	-	0.74	0.73	1
PdCuPtNiCo HEAs	PdCuPtNiCo nanoparticles	0.830	0.18	0.23	2
$iPd_3Pb@Pt/C$	$Pd_3Pb@Pt$ nanocubes	0.890	0.52	0.71	3
Pt-Cu-Mn UNFs	Ternary Pt-Cu-Mn nanoframes	-	1.45	3.38	4
$Pt_{37}Cu_{56}Au_7$	$Pt_{37}Cu_{56}Au_7$ porous film	0.909	0.87	1.85	5
Pt/MoN	Atomic Pt on MoN supports	-	0.71	-	6
Pd/Au-Pt NWs	Pd/Au-Pt core-shell nanowires	0.939	0.61	1.34	7
P-PtNi/C	P-doped PtNi concave octahedrons	0.900	0.70	1.65	8
$Pt_{75.4}Cu_{24.6}/GO$	$Pt_{75.4}Cu_{24.6}$ nanoparticles/GO	0.928	0.74	3.00	9
$Pd_{72}Pt_{28}$ NCs	$Pd_{72}Pt_{28}$ nanocubes	0.890	-	1.25	10
$Pt_{67}Cu_{33}$ NPs	PtCu nanoparticles	0.900	0.48	0.53	11
Pt_1-N/BP	Single atom Pt	-	0.70	-	12

- All of the potential values (V) were given with respective to reversible hydrogen electrode (RHE).
- The mass activity (MA) and specific activity (SA) of different catalysts were normalized at 0.9 V.

Supplementary Table 7. Pt-based core/shell catalysts towards ORR in 0.1 M $HClO_4$ electrolyte.

Catalysts	Morphology	Half-wave potential (V)	MA (A/mg_{Pt})	SA (mA/cm^2)	Reference
Pd_3Ru_1/Pt_{1-2L} NPs	PdRu/Pt core/shell nanoplates	0.930	4.59	3.16	This work
$L1_2$-Pt_3Co@ML-Pt/NPC_{10} NPs	Pt_3Co/Pt core/shell nanoparticles	0.940	1.72	3.49	13
Pt_3Ni@Ni-N_4-C	Pt_3Ni nanocages with Pt skin	-	1.92	2.65	14
Pd/PtFe NTs	Spiny Pd/PtFe core/shell nanotubes	0.930	2.71	4.32	15
fct-Pt-Co@Pt octahedra	PtCo/Pt core/shell octahedra	0.931	2.82	9.16	16
FePt@PtBi	FePt/PtBi core/shell nanoparticles	0.921	0.96	2.02	17
Pd/PdAu/Pt NWs	Pd/PdAu/Pt core/shell nanowires	0.930	1.54	1.15	18
PtNi-BNCs	PtNi nanocages with Pt skin	-	3.52	5.16	19
Pt-skin Pt_3Co NWs	Pt/Pt_3Co core/shell nanowires	-	2.20	5.60	20
Pt_3Fe z-NWs	Pt_3Fe nanowires with Pt skin	-	2.11	4.34	21
NPG-Pd-Pt	Nanoporous core/shell Au/PdPt	0.903	1.63	0.94	22
$PtNi_{1.21}Pd_{1.36}$ NWs	PtNiPd/Pt core/shell nanowires	-	1.93	3.48	23
$PtPb_{1.12}Ni_{0.14}$ octahedra	PtPb/PtNi core/shell octahedra	-	1.92	5.16	24
PtPb nanoplates	PtPb/Pt core/shell nanoplates	-	4.30	7.80	25
Pd@$Pt_{2.7L}$ icosahedra	Pd/Pt core/shell icosahedra	-	0.64	1.36	26
Octahedral PdPt nanocages	Pd/Pt core/shell nanocages	-	0.75	1.98	27

- All of the potential values (V) were given with respective to reversible hydrogen electrode (RHE).
- The mass activity (MA) and specific activity (SA) of different catalysts were normalized at 0.9 V.

References

1. Li, M., *et al*. Pt-Ni alloy nanoparticles via high-temperature shock as efficient electrocatalysts in the oxygen reduction reaction. *ACS Appl. Nano Mater.* **5**, 8243–8250 (2022).
2. Chen, Y., *et al*. Synthesis of monodisperse high entropy alloy nanocatalysts from core@shell nanoparticles. *Nanoscale Horiz.* **6**, 231–237 (2021).
3. Ashberry, H. M., Chen, C. & Skrabalak, S. E. Vertex-directed and asymmetric metal overgrowth of intermetallic Pd_3Pb@PtNi nanocubes for the oxygen reduction reaction. *ACS Appl. Nano Mater.* **4**, 12490–12497 (2021).
4. Qin, Y., *et al*. Fine-tuning intrinsic strain in penta-twinned Pt-Cu-Mn nanoframes boosts oxygen reduction catalysis. *Adv. Funct. Mater.* **30**, 1910107 (2020).
5. Xie, Y., *et al*. Enhanced ORR kinetics on Au-doped Pt-Cu porous films in alkaline media. *ACS Catal.* **10**, 9967–9976 (2020).
6. Li, S., *et al*. Impact of the coordination environment on atomically dispersed Pt catalysts for oxygen reduction reaction. *ACS Catal.* **10**, 907–913 (2019).
7. Chao, T., *et al*. Atomically dispersed Pt on screw-like Pd/Au core-shell nanowires for enhanced electrocatalysis. *Chemistry* **26**, 4019–4024 (2020).
8. Wang, S., *et al*. Structural and electronic stabilization of PtNi concave octahedral nanoparticles by P doping for oxygen reduction reaction in alkaline electrolytes. *ACS Appl. Mater. Interfaces* **10**, 27009–27018 (2018).
9. Liu, T., Li, C. & Yuan, Q. Facile synthesis of PtCu alloy/graphene oxide hybrids as improved electrocatalysts for alkaline fuel cells. *ACS Omega* **3**, 8724–8732 (2018).
10. Jukk, K., Kongi, N., Tammeveski, K., Solla-Gullón, J. & Feliu, J. M., Electroreduction of oxygen on PdPt alloy nanocubes in alkaline and acidic media. *ChemElectroChem* **4**, 2547–2555 (2017).
11. Zhao, Y., Wu, Y., Liu, J. & Wang, F. Dependent relationship between quantitative lattice contraction and enhanced oxygen reduction activity over Pt-Cu Alloy catalysts. *ACS Appl. Mater. Interfaces* **9**, 35740–35748 (2017).

12. Liu, J., *et al*. High-performance platinum single atom electrocatalyst for oxygen reduction reaction. *Nat. Commun.* **8**, 15938 (2017).

13. Wang, Z., *et al*. Tailored lattice compressive strain of Pt-skins by the $L1_2$-Pt_3M intermetallic core for highly efficient oxygen reduction. *Adv. Mater.* 2301310 (2023).

14. Yan, W., *et al*. Atomically dispersed Ni-N_4 sites assist Pt_3Ni nanocages with Pt skin to synergistically enhance oxygen reduction activity and stability. *Small* **19**, 2300200 (2023).

15. Tao, L., *et al*. Spiny Pd/PtFe core/shell nanotubes with rich high-index facets for efficient electrocatalysis. *Sci. Bull.* **66**, 44-51 (2021).

16. Xie, M., *et al*. Pt-Co@Pt octahedral nanocrystals: Enhancing their activity and durability toward oxygen reduction with an intermetallic core and an ultrathin shell. *J. Am. Chem. Soc.* **143**, 8509-8518 (2021).

17. Guan, J., *et al*. Intermetallic FePt@PtBi core-shell nanoparticles for oxygen reduction electrocatalysis. *Angew. Chem. Int. Ed.* **60**, 21899 (2021).

18. Tao, L., *et al*. Atomic PdAu interlayer sandwiched into Pd/Pt core/shell nanowires achieves superstable oxygen reduction catalysis. *ACS Nano* **14**, 11570-11578 (2020).

19. Tian, X., *et al*. Engineering bunched Pt-Ni alloy nanocages for efficient oxygen reduction in practical fuel cells. *Science* **366**, 850-856 (2019).

20. Luo, M., *et al*. Surface and near-surface engineering of PtCo nanowires at atomic scale for enhanced electrochemical sensing and catalysis. *Chem. Mater.* **30**, 6660-6667 (2018).

21. Luo, M., *et al*. Stable high-index faceted Pt skin on zigzag-like PtFe nanowires enhances oxygen reduction catalysis. *Adv. Mater.* **30**, 1705515 (2018).

22. Li, J., *et al*. Surface evolution of a Pt–Pd–Au electrocatalyst for stable oxygen reduction. *Nat. Energy* **2**, 17111 (2017).

23. Zhang, N., *et al*. Superior bifunctional liquid fuel oxidation and oxygen reduction electrocatalysis enabled by PtNiPd core-shell nanowires. *Adv. Mater.* **29**, 1603774 (2017).

24. Bu, L., *et al*. PtPb/PtNi intermetallic core/atomic layer shell octahedra for efficient oxygen reduction

electrocatalysis. *J. Am. Chem. Soc.* **139**, 9576-9582 (2017).

25. Bu, L., *et al*. Biaxially strained PtPb/Pt core/shell nanoplate boosts oxygen reduction catalysis. *Science* **354**, 1410-1416 (2016).
26. Wang, X., *et al*. Palladium-platinum core-shell icosahedra with substantially enhanced activity and durability towards oxygen reduction. *Nat. Commun.* **6**, 7594 (2015).
27. Zhang, L., *et al*. Platinum-based nanocages with subnanometer-thick walls and well-defined, controllable facets. *Science* **349**, 412-416 (2015).
